# How soundscape shapes restorative experience in a world heritage site: The mediating role of cultural authenticity at Mount Emei, China

**DOI:** 10.1371/journal.pone.0354957

**Published:** 2026-08-07

**Authors:** Haoran Wang, Muhammad Farid Azizul Azizui, Hao Liu, Jialan Hong, Peilei Zhou, Mengyun Yang

**Affiliations:** 1 Faculty of Built Environment and Surveying, Universiti Teknologi Malaysia, Johor Bahru, Malaysia; 2 School of Digital and Intelligent Design, Sichuan University of Culture and Arts, Mianyang, Sichuan, China; 3 School of Fashion Design, Jiangxi Institute of Fashion Technology, Nanchang, China; Zhejiang Agriculture and Forestry University: Zhejiang A and F University, CHINA

## Abstract

Sound plays an important role in heritage tourism experiences, yet limited research has examined how visitors’ positive perception of the sacred heritage soundscape supports restorative experience in World Heritage settings. This study investigated the relationships among positive heritage soundscape perception, cultural authenticity, and restorative experience at Mount Emei, a UNESCO World Heritage Site in China. Data were collected from 502 visitors across 10 sampling points using questionnaire surveys and on-site acoustic measurements. Structural equation modeling showed that visitors’ positive perception of the heritage soundscape was significantly and positively associated with restorative experience (*β* = 0.396, *p* < 0.001) and cultural authenticity (*β* = 0.405, *p* < 0.001). Cultural authenticity was also significantly and positively associated with restorative experience (*β* = 0.568, *p* < 0.001) and partially mediated the relationship between positive heritage soundscape perception and restorative experience (indirect effect *β* = 0.230, *p* < 0.001). The model explained 66.2% of the variance in restorative experience. Physical acoustic data were used to describe the objective sound conditions of the study site and to support the interpretation of the perceptual findings. The results suggest that positive evaluations of sacred heritage soundscapes support restoration through both a direct sensory pathway and an indirect pathway shaped by cultural authenticity.

## 1. Introduction

Modern urban life exposes people to constant stress, noise, and mental fatigue. Urban crowding, environmental noise, and continuous cognitive demands can reduce well-being and increase the need for restorative experiences [1]. In response, many people seek environments that help them relax, recover, and regain psychological balance. Natural and cultural landscapes have therefore become important destinations for leisure and restoration. Recent research has examined how multisensory experiences in historical and cultural settings can contribute to tourists’ mental restoration [2].

Acoustic conditions are a key part of visitors’ environmental experience. Earlier studies have shown that sounds from nature, including bird calls, running water, and wind, are often linked to lower stress and better recovery from cognitive fatigue [1,3]. These effects are commonly explained by two complementary theories. Attention Restoration Theory focuses on the recovery of depleted attentional resources, whereas Stress Reduction Theory emphasizes more immediate emotional and physiological recovery in supportive settings [4,5]. Although these perspectives have been widely applied in natural and nature-based recreational settings, including forests and rural landscapes [1,6–9], they have been used less often in heritage tourism environments. This gap is important because heritage settings often contain not only natural sounds, but also culturally meaningful sounds related to religion, ritual, and history.

The soundscape perspective offers a useful way to understand this issue. Under ISO 12913−1, soundscape is understood as the acoustic environment as people perceive or experience it within a particular context [10,11]. This definition highlights that soundscape is not only a physical condition, but also a subjective and contextual experience. Previous studies have shown that soundscape perception is mainly structured around the core dimensions of pleasantness and eventfulness [12,13]. At the same time, some researchers have suggested that additional descriptors, such as harmony, naturalness, or appropriateness, may help explain context-specific soundscape qualities in complex environments [14,15]. Empirical research further suggests that positively perceived soundscapes can improve emotional responses and psychological outcomes [6,7].

Recent scholarship also shows that heritage soundscapes should not be treated as static background conditions. Instead, they may form dynamic and meaningful experiential patterns across space. Jin et al. [16] showed, in their study of the Yungang Grottoes and Yongle Palace, that acoustic conditions may vary systematically across heritage zones and thereby influence visitors’ cultural and emotional responses. In addition, Hu et al. [17] showed that soundscape design in historic districts can strengthen authenticity by activating cultural memory, historical sense, and place identity. These findings suggest that in heritage settings, sound may influence visitor experience not only through sensory comfort, but also through cultural meaning.

Mount Emei, in Sichuan Province, China, is listed as a UNESCO World Heritage Site and is widely valued for its outstanding natural landscape and long-standing Buddhist tradition [18]. The close integration of natural and cultural features within this setting makes it especially suitable for examining how visitors’ positive perception of the heritage soundscape relates to cultural and restorative experience. In particular, Mount Emei provides a valuable context for exploring the links among positive heritage soundscape perception, cultural authenticity, and restorative experience.

Cultural authenticity is an important concept in heritage tourism research. Authenticity refers to whether tourists perceive cultural elements at a destination as genuine and meaningful [19,20]. Previous studies distinguish between objective authenticity, which concerns the genuineness of cultural objects and settings, and existential authenticity, which concerns visitors’ inner feelings of self-connection and meaningful engagement during travel [20]. In heritage environments, authenticity is often discussed in relation to visual and architectural elements. However, recent studies suggest that sound can also function as a powerful cultural cue. Sounds such as chanting, temple bells, and ritual activities may communicate sacredness, continuity, and local identity [17]. This means that cultural authenticity may help explain how visitors interpret heritage soundscapes.

Restorative experience refers to the process through which people recover from mental fatigue and psychological stress in supportive environments. Attention Restoration Theory proposes that restorative environments help restore attentional resources [4], while Stress Reduction Theory emphasizes rapid emotional and physiological recovery from stress [5,21]. Although restoration has been examined mainly in natural environments, less attention has been given to heritage settings where acoustic experience is closely connected with religious and cultural meanings. Recent research also suggests that emotionally meaningful environments may support restoration through deeper psychological pathways, such as emotional bonding with place [6]. This indicates that restoration in heritage sites may involve more than sensory comfort alone.

Although the restorative benefits of natural sounds have been widely documented, less is known about how restorative experience is formed in sacred heritage settings where acoustic experience is intertwined with pilgrimage, religious atmosphere, cultural memory, and place identity. In such contexts, visitors’ positive perception of the heritage soundscape may not operate only as a sensory stimulus. It may also function as a cultural cue that helps visitors evaluate the authenticity of the site. Therefore, the present study does not merely test whether pleasant soundscapes are associated with restoration; rather, it examines whether cultural authenticity explains the relationship between positive heritage soundscape perception and restorative experience in a religious World Heritage context.

To address this gap, the present study examines how visitors’ positive perception of the heritage soundscape at Mount Emei is associated with restorative experience, and whether cultural authenticity mediates this relationship. Structural equation modeling (SEM) was employed to test the proposed framework. The study contributes to the literature in several ways. It extends soundscape research to a World Heritage context, explains the meaning-based mediating role of cultural authenticity between positive heritage soundscape perception and restorative experience, and offers practical implications for heritage site managers concerned with soundscape protection and design. Although restorative experience in sacred heritage settings is shaped by multiple psychological, cultural, and tourism-related factors, the present study specifically focuses on the role of positive heritage soundscape perception as an important experiential component within this broader context.

## 2. Literature review

### 2.1 Positive heritage soundscape perception

The idea of soundscape can be traced back to R. Murray Schafer, who emphasized the acoustic environment as something heard and interpreted by people [22]. This idea was eventually defined in ISO 12913−1, which defines soundscape as the acoustic environment as something heard and experienced by people [10,11]. This definition indicates that soundscape research goes beyond conventional noise assessment. It considers not only physical sound levels, but also the ways in which people interpret and evaluate sound in a given setting.

One major development in this field concerns the perceptual structure of soundscape. Axelsson et al. [12] proposed that pleasantness and eventfulness are the two main dimensions of soundscape perception. This two-dimensional structure has been widely accepted and later supported by the ISO 12913 framework. At the same time, some studies have argued that additional descriptors may be useful in special settings. Aletta et al. [14] noted that descriptors such as appropriateness to place can complement the core dimensions. Similarly, Hong and Jeon [13] showed that contextual and acoustic factors jointly shape soundscape evaluation. In the present study, harmony is retained as a context-sensitive dimension. It is used to reflect the perceived fit and coherence among natural sounds, cultural sounds, and the sacred heritage setting of Mount Emei, rather than as a replacement for the core ISO-based dimensions.

Previous studies consistently show that different sound types produce different perceptual and psychological responses. Sounds from nature, such as birdsong, running water, and wind are generally evaluated more positively, whereas traffic noise and intrusive human-made sounds are more often associated with annoyance and lower environmental quality [7,23]. These results suggest that soundscape perception is closely related to emotional and environmental evaluation. However, in heritage settings, sound has an added layer of cultural meaning. Sounds may function not only as sensory stimuli, but also as carriers of memory, ritual, identity, and symbolic meaning.

Recent studies have increasingly examined soundscape in cultural heritage contexts. Hu et al. [17] found that soundscape design in historic districts can strengthen authenticity by activating historical sense, cultural memory, and intangible heritage value. Mao et al. [24] showed that both natural and cultural soundscapes positively influenced visitors’ environmental behavior in the Grand Canal National Cultural Park in China. Jin et al. [16] further showed, in their study of the Yungang Grottoes and Yongle Palace, that heritage soundscapes are not evenly distributed. Instead, they may form a spatial acoustic gradient across different site zones. This acoustic sequence can shape cultural-religious experience, visitor identity, and loyalty. These findings suggest that heritage soundscapes differ from those of ordinary recreational spaces because they combine sensory quality with cultural significance.

Soundscape studies have also advanced methodologically. Questionnaire surveys, semantic differential scales, and in-situ acoustic measurements are commonly used to assess soundscape perception [25,26]. Soundwalks are also widely used because they allow researchers to capture visitors’ real-time responses while moving through a site [26]. Given the mixed natural and religious sound sources at Mount Emei, these approaches provide a useful methodological basis for examining how visitors perceive the site’s acoustic environment.

### 2.2 Cultural authenticity in heritage tourism

Cultural authenticity is widely regarded as a central concept in heritage tourism research. Early studies distinguished between objective authenticity and constructed authenticity, emphasizing that authenticity may refer either to the genuineness of cultural objects or to socially negotiated interpretations of what is considered authentic [19]. Wang [20] later expanded on this conversation by proposing existential authenticity, which refers to a state in which travelers feel more connected to themselves and the cultural surroundings when traveling. From this perspective, authenticity is not limited to whether an object is historically original, but also involves the emotional and experiential dimensions of tourism.

In heritage tourism contexts, cultural authenticity has been linked to beneficial tourist outcomes, including destination loyalty, well-being, and memorability [27,28]. When tourists see a destination’s cultural features as authentic and important, they are more likely to report richer and more memorable experiences. Authenticity therefore functions not only as a judgment about heritage objects, but also as a psychological evaluation that shapes how visitors respond to a destination.

Although authenticity has been widely studied in relation to heritage objects, cultural identity, visitor engagement, and heritage consumption [19,20,29,30], the role of sound in shaping authenticity perception remains relatively underexplored. Yet in many heritage environments, sound functions as an important cultural cue. Temple bells, chanting, ritual sounds, and other acoustically embedded cultural elements can communicate continuity, sacredness, and local identity. Hu et al. [17] showed that carefully designed soundscapes can improve visitors’ perception of authenticity in historic districts. In religious heritage settings such as Mount Emei, where natural and Buddhist sounds coexist, the acoustic environment may therefore play a direct role in shaping whether visitors perceive the site as culturally genuine and meaningful.

In religious heritage tourism, visitors’ restorative responses may also be shaped by pilgrimage motives, spiritual expectations, prior religious knowledge, and cultural familiarity. These factors may influence how visitors interpret temple bells, chanting, ritual music, and other religious sounds. Studies of religious historical buildings, historically significant places, and heritage authenticity suggest that sacred environments are experienced through intertwined sensory, cultural, and psychological meanings [31–33]. This discussion reflects a broader debate in religious heritage tourism research: religious heritage experience should not be understood as a direct response to environmental stimuli alone, but as an experience constructed through the interaction between sensory cues, cultural knowledge, religious atmosphere, personal motivation, and visitors’ familiarity with the heritage context [46]. Therefore, soundscape perception should be understood as one component of a broader religious and cultural experience rather than as the sole determinant of restoration. This point is particularly important at Mount Emei, where natural soundscapes, Buddhist cultural meanings, and visitor motivations are closely intertwined.

At the same time, authenticity should not be treated as a completely uniform concept. Objective authenticity may be more closely related to judgments about whether cultural elements are original and faithful to tradition. Existential authenticity, in contrast, may be more closely related to immersion, self-connection, emotional release, and meaningful engagement [20]. This distinction is important because different forms of authenticity may influence visitor outcomes in different ways. In the context of Mount Emei, visitors may cognitively recognize a bell or chant as culturally authentic, but deeper restorative experience may depend more on whether such sounds also create emotional resonance and a sense of inner connection. Although the present study models Cultural Authenticity as an integrated construct, this conceptual distinction remains important for interpreting the findings.

Despite its theoretical importance, Cultural Authenticity has rarely been examined as a mediating mechanism linking environmental perception to restorative outcomes. Existing studies have examined its relationships with destination loyalty, well-being, and memorability [27,28]. Much less attention has been given to its role in explaining how heritage soundscapes contribute to restorative experience. This gap provides an important basis for the present study.

### 2.3 Restorative experience and theoretical foundations

Restorative experience describes the process through which people regain psychological resources in supportive environments, including recovery from mental fatigue, attentional exhaustion, and stress. This process is commonly interpreted through two related theoretical perspectives: Attention Restoration Theory and Stress Reduction Theory.

Attention Restoration Theory, introduced by Kaplan and Kaplan [3] and further developed by Kaplan [4], explains restoration primarily in terms of recovery from directed attention fatigue. Within this framework, restorative environments are usually described through four qualities: being away, fascination, extent, and compatibility. Being away refers to a psychological distance from daily responsibilities. Fascination refers to effortless attention drawn by interesting stimuli. Extent reflects the perception of being in a coherent and sufficiently rich environment, while compatibility refers to the fit between environmental affordances and personal needs. These four dimensions have been widely used to assess restorative experience in environmental psychology research [34,35].

Stress Reduction Theory, proposed by Ulrich [21] and Ulrich et al. [5], complements Attention Restoration Theory by focusing on more immediate emotional and physiological responses to supportive environments. According to this perspective, exposure to certain environments can rapidly reduce stress, lower physiological arousal, and promote positive affect. While Attention Restoration Theory emphasizes attentional recovery, Stress Reduction Theory highlights emotional calmness and stress relief. Together, the two theories provide a comprehensive explanation of why certain environments are experienced as restorative.

Attention Restoration Theory and Stress Reduction Theory have been widely used in studies of natural environments, including forests, urban parks, and waterfront areas. More recently, soundscape research has begun to adopt these frameworks to explain how acoustic environments contribute to restoration [7,23]. Natural sounds have been found to support relaxation, reduce stress, and enhance restorative perceptions, whereas traffic and intrusive artificial noise tend to undermine these effects. However, most existing studies focus on natural recreational settings [1,8]. Comparatively little is known about how restorative experience is generated in heritage environments where acoustic experience is intertwined with cultural and symbolic meaning.

This limitation is important because heritage environments may support restoration not only through sensory comfort, but also through interpretation, immersion, and cultural engagement. In sacred heritage sites, sounds may operate as both restorative stimuli and carriers of cultural meaning. This suggests that restorative experience in such settings may involve more complex mechanisms than those identified in conventional natural-environment studies.

### 2.4 The relationship between positive heritage soundscape perception, cultural authenticity, and restorative experience

Although positive heritage soundscape perception, understood here as visitors’ evaluation of pleasant, harmonious, and contextually meaningful acoustic environments, has been linked to psychological restoration, and cultural authenticity has been widely examined in heritage tourism, few studies have explored how these two constructs interact, particularly in World Heritage Sites. Most soundscape studies focus on natural parks and urban green spaces [6,7,9], while authenticity research mainly examines visitor satisfaction and behavioral intention [20,28]. The pathway through which positive heritage soundscape perception shapes cultural authenticity and subsequently leads to restorative experience has rarely been examined empirically. This gap is important because heritage sites often possess distinctive acoustic environments in which natural sounds and culturally meaningful sounds coexist and convey symbolic meaning.

The first step of the proposed mechanism concerns how positive heritage soundscape perception is associated with cultural authenticity. Sounds such as temple bells, ritual chanting, and traditional music often signal the historical and cultural identity of heritage places. When visitors find the soundscape pleasant and harmonious, they are more inclined to consider the place as culturally authentic. This association has been supported by empirical studies. Hu et al. [17] showed that soundscape design can strengthen perceived authenticity in historic districts. Jin et al. [16] showed that heritage soundscapes may shape visitors’ cultural-religious experience, identity, and loyalty across different heritage zones. These findings suggest that meaningful soundscapes do not only improve acoustic comfort but also communicate cultural continuity and symbolic meaning, which are central elements of authenticity perception.

The second step of the mechanism concerns how cultural authenticity contributes to restorative experience. Wang [20] stated that existential authenticity refers to a psychological condition in which tourists feel more connected to themselves and to their cultural surroundings. This interpretation is also consistent with research on visitor engagement and authenticity in heritage consumption [29]. This state is closely related to the restorative conditions described in Attention Restoration Theory [4]. Visitors are more likely to feel removed from the demands of daily life and to engage with the surroundings effortlessly when they see a cultural setting as authentic and relevant. Authentic cultural sounds, such as chanting or temple bells, may naturally attract attention and encourage immersion. Previous research shows that culturally meaningful sensory environments can foster emotional resonance and support psychological recovery [36].

Taken together, these two steps suggest that cultural authenticity functions as a psychological bridge between positive heritage soundscape perception and restorative experience. Meaningful soundscapes may strengthen visitors’ perception that a heritage environment reflects genuine cultural traditions. This perception of authenticity can then deepen emotional engagement and support restorative processes. Although previous studies have examined individual relationships between positive soundscape perception and restoration [1,7] and between authenticity and visitor experience [28], the full pathway linking positive heritage soundscape perception, cultural authenticity, and restorative experience has rarely been tested within a single empirical model. The current study closes this gap by evaluating an integrated framework in which positive heritage soundscape perception is treated as an environmental appraisal, cultural authenticity as a meaning-based interpretive mechanism, and restorative experience as the psychological outcome.

### 2.5 Hypothesis development

#### 2.5.1 Positive heritage soundscape perception and cultural authenticity.

In this study, soundscape perception does not refer to sound perception in a general or neutral sense. It refers specifically to visitors’ positive evaluation of the heritage soundscape in context, reflected by perceived pleasantness, harmony, and eventfulness. Such evaluations are not limited to the physical properties of sound, but also reflect how visitors interpret environmental meaning through sound [12]. In heritage settings, sounds may function as cultural cues that signal local identity, collective memory, religious atmosphere, and historical continuity. Research in historical and cultural areas has shown that meaningful soundscapes can evoke culture and memory, strengthen emotional connection to place, and shape sense of place [37]. In the present study, eventfulness is not treated as a simple indicator of acoustic busyness or noise intensity. Rather, it refers to visitors’ perception that the heritage soundscape contains meaningful and contextually appropriate sound events, such as temple bells, chanting, flowing water, and ritual ambience, which contribute to the perceived vitality of the sacred heritage setting.

This logic is particularly pertinent at Mount Emei. Visitors hear not only natural sounds, but also culturally significant ones like temple bells and Buddhist chanting. When these sounds are perceived as pleasant, harmonious, and appropriate to the setting, visitors may be more likely to interpret the site as genuine and culturally meaningful. In heritage tourism, authenticity has been recognized as a central evaluative construct linking cultural meaning to visitor responses [30]. Therefore, this study proposes:

**H1:** Visitors’ positive perception of the heritage soundscape, reflected by pleasantness, harmony, and eventfulness, is positively associated with perceived cultural authenticity.

#### 2.5.2 Cultural authenticity and restorative experience.

Cultural authenticity refers to the degree to which visitors view the cultural features of a heritage site as genuine, meaningful, and congruent with the identity of the place. In the present model, cultural authenticity is not treated as a final outcome equivalent to restorative experience. Rather, it is conceptualized as a meaning-based appraisal through which visitors interpret the heritage environment. When tourists perceive the surrounding environment as culturally authentic, they may become more deeply engaged with the place. This engagement can create psychological conditions that support restoration. According to Attention Restoration Theory, restorative surroundings help recovery from mental fatigue through being away, fascination, extent, and compatibility [4]. These restorative conditions may arise not only in natural settings, but also in culturally meaningful environments that promote immersion and coherence.

Previous studies suggest that authenticity is positively related to psychological and emotional outcomes in heritage tourism. Yi et al. [28] demonstrated that authenticity is positively connected with well-being, whereas Zhou et al. [2] showed that multimodal cultural experiences in historic blocks can boost mental healing. In the context of Mount Emei, when visitors see the site as culturally authentic, they may be more inclined to feel psychologically removed from routine demands, more effortlessly engaged with the environment, and more compatible with the setting. Therefore, this study proposes:

H2: Perceived cultural authenticity of the sacred heritage setting is positively associated with visitors’ restorative experience.

#### 2.5.3 Direct association between positive heritage soundscape perception and restorative experience.

Visitors’ positive perception of the heritage soundscape may also be directly associated with restorative experience. Previous research in restorative environments has shown that natural sounds and positively evaluated soundscapes can support recovery from stress and mental fatigue. Ratcliffe [1] concluded that sounds such as birdsong, water, and wind can enhance restorative outcomes and improve positive environmental evaluations. Empirical research also supports a direct relationship between soundscape evaluation and restoration. Bai et al. [38] found that soundscape perception in an ancient town was significantly associated with tourism experiences, while Yang and Zhang [9] showed that rural soundscape perception had a significant positive association with environmental restoration perception.

This direct pathway is included not because the association between pleasant soundscapes and relaxation is assumed to be novel in itself, but because it provides a necessary comparison with the indirect cultural-authenticity pathway. Acoustic stimuli such as flowing water or birdsong can attract attention without mental effort, reduce stress, and help visitors distance themselves from routine demands. At Mount Emei, natural sound elements may therefore contribute directly to restorative experience even before visitors interpret the environment in explicitly cultural terms. Estimating this direct path allows the study to distinguish an immediate sensory-restorative association from a meaning-based pathway mediated by cultural authenticity. Therefore, this study proposes:

**H3:** Visitors’ positive perception of the heritage soundscape is positively associated with restorative experience.

#### 2.5.4 The mediating role of cultural authenticity.

The central argument of this study is that cultural authenticity mediates the link between positive heritage soundscape perception and restorative experience. In a sacred heritage setting, visitors’ positive evaluation of the soundscape may first strengthen their perception that the environment is culturally genuine, and this stronger perception of authenticity may then contribute to a deeper restorative experience.

This mediation logic is supported by related research showing that soundscape does not always influence psychological outcomes through direct sensory evaluation alone. Intermediate psychological mechanisms can also play an important role. For example, Yang and Zhang [9] found that rural soundscape perception was linked to environmental restoration through tourism nostalgia and place attachment, suggesting that restorative associations can be transmitted through cognitive and emotional processes. In a World Heritage Site, cultural authenticity is likely to be a more contextually appropriate mediator because visitors respond not only to comfort and calmness, but also to whether the environment reflects the true cultural identity of the site. Therefore, this study proposes:

**H4:** Cultural authenticity mediates the association between visitors’ positive perception of the heritage soundscape and restorative experience.

#### 2.5.5 Model construction.

**Fig 1:**
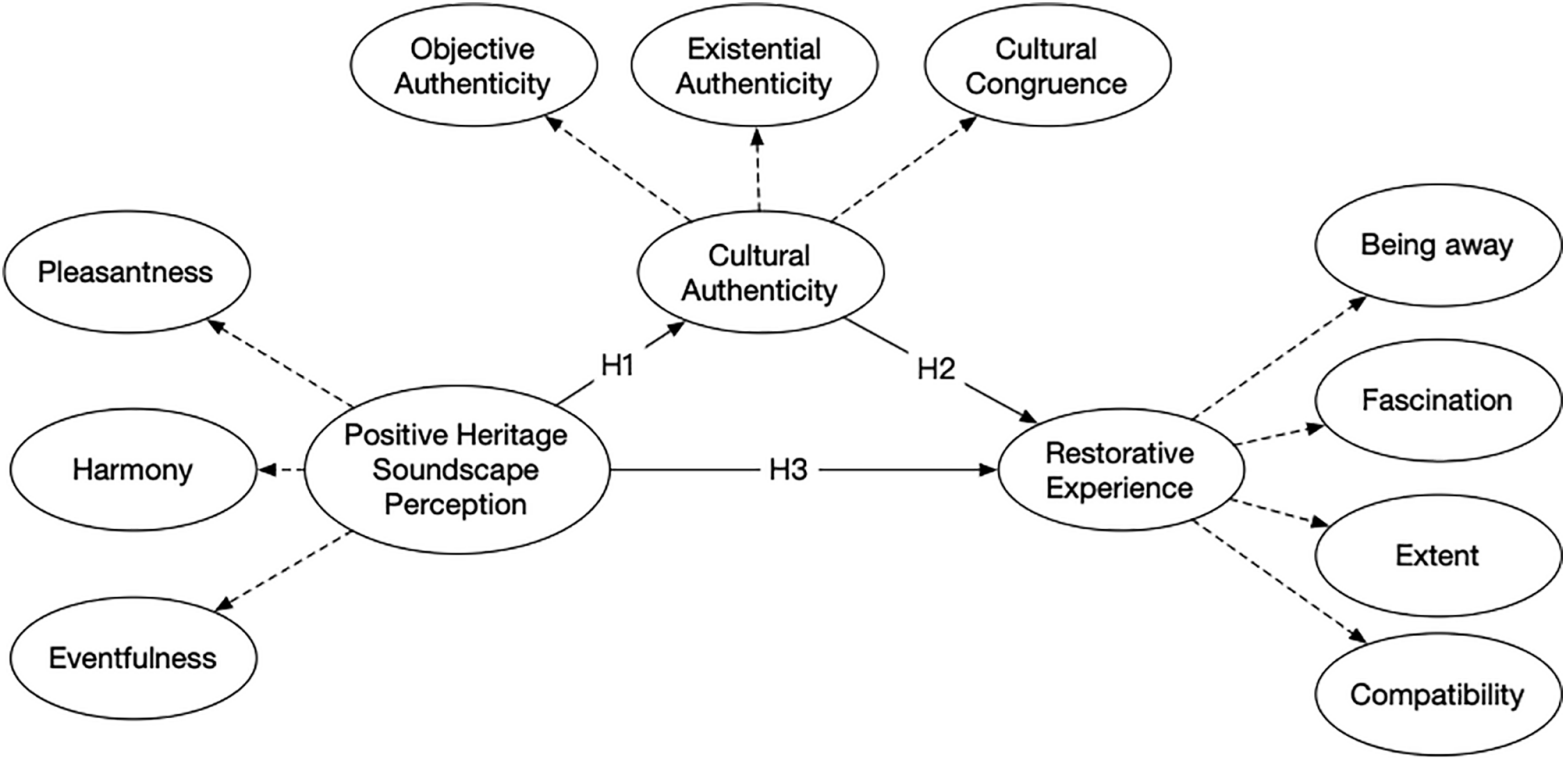
Proposed conceptual model. Source: Authors’ conceptual model developed from soundscape perception theory [8,10], authenticity theory [19,20,30], and restoration theory [4,5].

This study develops a conceptual model to investigate the connections between positive heritage soundscape perception, cultural authenticity, and restorative experience in the setting of a World Heritage Site. The model does not treat the three constructs as equivalent outcome variables. Instead, positive heritage soundscape perception is conceptualized as visitors’ appraisal of acoustic environmental cues, cultural authenticity as a meaning-based interpretive mechanism, and restorative experience as the psychological outcome. As shown in Fig 1, positive heritage soundscape perception is proposed to be associated with restorative experience both directly and indirectly through cultural authenticity. In the model, positive heritage soundscape perception is conceptualized as a second-order construct reflected by pleasantness, harmony, and contextually meaningful eventfulness. Cultural authenticity is measured through objective authenticity, existential authenticity, and cultural congruence. Restorative experience is operationalized through four dimensions derived from Attention Restoration Theory, namely being away, fascination, extent, and compatibility. Fig 1 is the authors’ conceptual model developed from soundscape theory [8,10], authenticity theory [19,20,30], and restoration theory [4,5].

## 3. Methodology

### 3.1 Study site

Mount Emei Scenic Area, which also includes the Leshan Giant Buddha Scenic Area, was added to the UNESCO World Heritage List in 1996. The site is recognized for both its natural value and its long Buddhist history, which makes it an appropriate setting for the present study [18]. As the specific study site, Mount Emei is particularly suitable because its acoustic environment contains both natural sounds, such as birdsong, mountain streams, and wind through the forest, and culturally significant sounds, such as temple bells, chanting, and other ritual activities. The coexistence of these sound sources creates a distinctive acoustic setting, providing a strong context for examining the relationships among positive heritage soundscape perception, cultural authenticity, and restorative experience.

**Fig 2:**
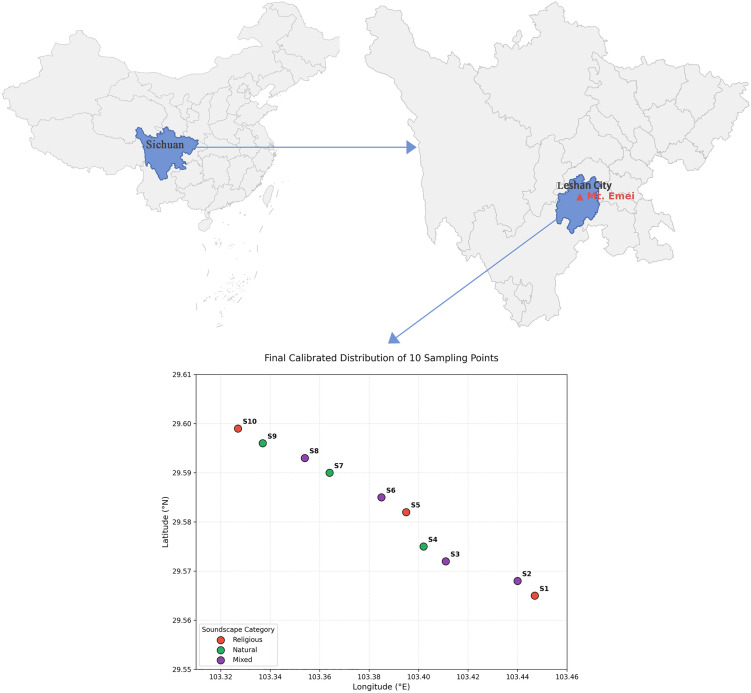
Location of the study area and spatial distribution of the 10 sampling points in Mount Emei Scenic Area, China. The nested maps show the location of Sichuan Province in China and Leshan City in Sichuan. The main map shows the sampling-point coordinates and soundscape categories. The figure was created by the authors using authors’ sampling-point coordinates and public-domain Natural Earth boundary data; no proprietary map, satellite, Google Maps, Google Earth, or Street View imagery was used.

To capture variation in acoustic conditions across the scenic area, ten sampling points were selected along the main tourist route. These points represented three broad soundscape contexts, namely natural, religious, and mixed soundscape zones. The study area and spatial distribution of the sampling points are shown in Fig 2, and the sampling points, dominant sound sources, acoustic levels, and soundscape categories are summarized in Table 1.

**Table 1:** Sampling points and soundscape categories in Mount Emei.

Code	Sampling point	Soundscape category	Dominant sound sources	LAeq [dB(A)]	Notes
S1	Baoguo Temple	Religious	Temple bells, chanting, ritual sounds	54.2	Low altitude (550m); high crowd flow
S2	Fuhu Temple	Mixed	Wind in forest, temple sounds, bird calls	48.5	Dense forest buffer; quiet ritual sounds
S3	Qingyin Pavilion	Mixed	Streams, water sounds, visitor voices	61.8	Rushing water from dual rivers (Loudest)
S4	Ecological monkey area	Natural	Stream sounds, birdsong, wind, monkey calls	58.4	Continuous stream noise & visitor activity
S5	Wannian Temple	Religious	Buddhist music, chanting, temple ambience	52.6	Mid altitude (1020m); chanting & bells
S6	Hongchunping	Mixed	Rain, water sounds, birdsong, quiet temple sounds	46.7	Misty environment; low background noise
S7	Jiulinggang trail	Natural	Footsteps, wind, birdsong	43.5	Remote hiking trail; birdsong (Quietest)
S8	Xixiangchi	Mixed	Wind, evening chanting, mountain ambience	49.2	High altitude (2070m); wind & chanting
S9	Leidongping to Jieyin Hall trail	Natural	Forest sounds, wind, birdsong	47.8	High altitude forest; dominated by wind
S10	Golden Summit	Religious	Ritual sounds, chanting, mountain wind	56.3	Summit (3079m); strong wind & large crowds

### 3.2 Data collection and sampling procedure

Data were collected through on-site acoustic measurement and a questionnaire survey. The fieldwork and participant recruitment were conducted from 13 October 2025–8 November 2025. Both procedures were conducted at the same sampling points during the same field sessions in order to ensure consistency between the measured acoustic conditions and visitors’ perceptual responses. Acoustic data were recorded using a calibrated sound level meter. At each point, the A-weighted equivalent continuous sound pressure level (LAeq) was recorded over a 10-minute period as part of the on-site soundscape data collection procedure, following ISO/TS 12913−2 [26]. During each measurement period, dominant sound sources were also recorded by trained observers. The sampling points covered low-, mid-, and high-altitude areas and represented three broad soundscape categories: natural, religious, and mixed. The sampling points and their main acoustic characteristics are summarized in Table 1. These LAeq values were used to describe the objective sound conditions across the study site and to provide contextual support for interpreting the perceptual results.

The questionnaire survey adopted the soundwalk approach recommended in ISO 12913−2 for outdoor soundscape research. Visitors were invited to complete the survey after spending at least five minutes at a sampling point so that they had sufficient exposure to the local sound environment. Convenience sampling was used because of the practical constraints of visitor recruitment in a World Heritage tourism setting. Surveys were administered in Mandarin Chinese on both weekdays and weekends to capture a larger range of visitor experiences. Following the soundwalk procedure, respondents were instructed to focus on the surrounding acoustic environment before completing the questionnaire.

Paper-based or QR-code-based questionnaires were distributed at each sampling point by four trained research assistants after visitors had stayed at or passed through the local sound environment for at least five minutes. The survey was administered at the sampling points rather than only at the entrance, so that responses could be linked to the corresponding acoustic context. Data collection was conducted during daytime visiting hours on both weekdays and weekends. The main route covered low-, middle-, and high-altitude sections of the scenic area. Because respondents were recruited at individual sampling points rather than asked to complete the entire route, the analysis focused on site-specific acoustic exposure rather than full-route walking experience.

The survey was administered in Mandarin Chinese because the study focused on domestic visitors’ perceptions of Mount Emei and because domestic tourists constitute the main visitor group at the site. International visitors were not included because language differences, cultural familiarity with Buddhist heritage, and different travel motivations could introduce additional heterogeneity beyond the scope of the present study.

Before starting the survey, participants were presented with a clear informed consent statement on the first page of the questionnaire. The statement explained the study purpose, voluntary participation, anonymity, confidentiality, and the right to withdraw. Written/electronic informed consent was obtained by asking participants to tick a consent box before proceeding to the questionnaire. No signatures or personal identifiers were collected in order to maintain anonymity. Only participants who provided informed consent were allowed to continue. All participants were adults aged 18 years or above; therefore, parental or guardian consent was not required. The study was conducted in accordance with the Declaration of Helsinki. Ethical review and approval were waived for this study because it involved an anonymous questionnaire, did not collect personally identifiable or sensitive information, posed no foreseeable harm to individuals, and was not associated with commercial interests. The legal basis for waiving ethical review and approval is Article 32 of the Ethical Review Measures for Life Sciences and Medical Research Involving Humans, promulgated by the National Health Commission of China and published on 18 February 2023 (https://www.gov.cn/zhengce/zhengceku/2023-02/28/content_5743658.htm).

Before the formal survey, a pilot study involving 30 participants was carried out to assess the clarity of the items and the internal consistency of the scale. Based on the pilot feedback, several minor wording adjustments were introduced, and the pilot data were not included in the final analysis.

In total, 725 questionnaires were distributed and 584 were returned, giving a response rate of 80.55%. After data screening, 502 questionnaires were judged to be valid and were retained for analysis. This corresponds to a valid response rate of 69.24% relative to the total number distributed. The demographic profile of the valid respondents is presented in Table 2.

**Table 2:** Demographic profile of respondents.

Demographic variable	Category	Frequency	Percentage (%)
Gender	Male	237	47.21%
	Female	265	52.79%
Age	18-25	135	26.89%
	26-35	125	24.90%
	36-45	160	31.87%
	46-55	68	13.55%
	56 and above	14	2.79%
Education level	High school or below	43	8.57%
	Junior college	185	36.85%
	Bachelor’s degree	215	42.83%
	Graduate degree or higher	59	11.75%
Occupation	Student	96	19.12%
	Businessman	88	17.53%
	Private employee	138	27.49%
	Government employee	35	6.97%
	Unemployed	46	9.16%
	Others	99	19.72%
Individual monthly income	1500 and below	45	8.96%
	1501–3000	89	17.73%
	3001–5000	128	25.50%
	5001–8000	156	31.08%
	8001–15000	32	6.37%
	Above 15000	52	10.36%

The anonymized dataset and accompanying codebook are provided in S1 Data, while the bilingual measurement items are provided in S1 Table. The dataset contains no direct personal identifiers and includes only the variables necessary to reproduce the descriptive statistics, reliability and validity tests, SEM results, and robustness checks.

### 3.3 Measures

The questionnaire was organized into four sections. The first section gathered demographic information, while the remaining three sections measured positive heritage soundscape perception, cultural authenticity, and restorative experience. All items were assessed using a seven-point Likert scale, with response options ranging from 1 (“strongly disagree”) to 7 (“strongly agree”).

Positive heritage soundscape perception was modeled as a second-order construct represented by pleasantness, harmony, and eventfulness. The items were adapted from ISO 12913−2 [26] and the soundscape perception model proposed by Axelsson et al. [12]. Cultural authenticity was conceptualized as a second-order construct reflected by objective authenticity, existential authenticity, and cultural congruence. Since no established scale directly captured heritage soundscape authenticity in the present context, these items were developed for this study based on the theoretical frameworks of Cohen [19] and Wang [20], and were refined through expert review and pilot testing. Restorative experience was conceptualized as a second-order construct reflected by being away, fascination, extent, and compatibility. These items were adapted from restoration research grounded in Attention Restoration Theory, especially Hartig et al. [39] and Qiu et al. [40]. A summary of the constructs, dimensions, and measurement sources is presented in Table 3. The full item list is provided in S1 Table.

**Table 3:** Summary of constructs, dimensions, item numbers, and measurement sources.

Construct	Dimension	No. of items	Source
Positive Heritage Soundscape Perception	Pleasantness	3	[12,26]
	Harmony	3	
	Eventfulness	3	
Cultural Authenticity	Objective Authenticity	3	[19,20]
	Existential Authenticity	3	
	Cultural Congruence	3	
Restorative Experience	Being away	4	[39,40]
	Fascination	4	
	Extent	4	
	Compatibility	4	

Besides the questionnaire survey, objective acoustic measurements were also taken at each sampling point. The LAeq was recorded to describe the physical acoustic conditions of each location. In the present study, these acoustic data were used as contextual environmental information to support site description and result interpretation, rather than being specified as a structural variable in the SEM model.

Acoustic measurements were used to characterize the objective acoustic environment of each sampling point and to contextualize visitors’ perceptual responses. Because LAeq was measured at the site level, whereas the SEM focused on individual-level perceptions, LAeq was not specified as a latent predictor in the structural model. To address the site-level nature of the acoustic data, additional descriptive comparisons across sampling points and soundscape categories were added to the Results section.

### 3.4 Common method bias assessment

Because all latent variables were measured through self-reported questionnaire responses collected in the same survey, common method bias was examined before model evaluation. Following Harman [41], a single-factor test was applied by loading all measurement items into an unrotated exploratory factor analysis. The first unrotated factor accounted for 31.708% of the total variance, which was lower than the commonly used threshold of 50%. However, because Harman's single-factor test is recognized as a preliminary and limited diagnostic rather than a definitive test of common method variance [42], this result was interpreted cautiously. On this basis, common method bias was not considered to pose a serious problem in the present study, although this limitation is acknowledged.

To further control and assess common method bias (CMB) with greater statistical rigor, an unmeasured latent method construct (ULMC) was incorporated into the measurement model [42], allowing all measurement items to load simultaneously on their respective substantive factors and the common method factor. A comparison of the standardized factor loadings before and after the inclusion of the method factor revealed that the variations in standardized loadings were minimal, with all changes below the empirical threshold of 0.20 [43]. These statistical diagnostics consistently suggest that common method bias does not pose a serious threat to this study and is unlikely to substantially distort the structural relationship estimates.

### 3.5 Data analysis

Before estimating the structural equation model, the distributional characteristics of the observed variables were examined to assess whether the data were appropriate for covariance-based structural equation modeling (CB-SEM). The CB-SEM model was estimated using SmartPLS 4.0. As shown in Table 4, the univariate skewness values for all first-order constructs ranged from −0.082 to 0.213, while the univariate kurtosis values ranged from −0.586 to 0.203. These values were well within the commonly accepted thresholds of |3| for skewness and |10| for kurtosis [44], indicating that the observed variables did not exhibit severe departures from normality. CB-SEM was selected rather than PLS-SEM because the present study aimed to test a theory-driven reflective measurement model and evaluate overall model fit, rather than primarily maximize prediction. The sample size and distributional characteristics were also considered suitable for CB-SEM estimation.

**Table 4:** Assessment of normality.

Assessment	Value / Range	Criterion	Interpretation
Univariate skewness	−0.082 to 0.213	|skewness| < 3	Acceptable
Univariate kurtosis	−0.586 to 0.203	|kurtosis| < 10	Acceptable
Mardia’s multivariate kurtosis	z = -1.480, p = 0.139	p > 0.05	Acceptable

In addition to univariate normality, Mardia’s multivariate kurtosis was also examined to evaluate the multivariate distribution of the data. As presented in Table 4, the multivariate kurtosis test did not indicate severe multivariate non-normality (z = −1.480, p = 0.139). Taken together, these results suggested that the data were acceptable for CB-SEM estimation. Furthermore, bootstrap-based confidence intervals were calculated in SmartPLS for the mediation analysis to provide more robust statistical inference and to reduce reliance on strict distributional assumptions.

Overall model fit was assessed using χ², df, χ²/df, CFI, TLI, RMSEA, and SRMR. The structural model was then examined using standardized path coefficients, significance levels, and explained variance (*R²*). The mediating association of cultural authenticity was assessed through the indirect association and its 95% confidence interval. Because the model included reflective higher-order constructs, one loading for each first-order factor was fixed to 1.0 to ensure model identification.

To further examine the stability of the proposed structural relationships, subgroup robustness checks were conducted by gender and age groups. These analyses were used to assess whether the main path directions remained generally stable across different demographic groups.

## 4. Results

### 4.1 Descriptive statistics and acoustic characteristics

Before the measurement model assessment, descriptive statistics were examined for all first-order dimensions. As shown in Table 5, the mean values ranged from 3.273 to 3.693, while the standard deviations ranged from 0.820 to 0.992. These results indicate that the Likert-scale responses showed sufficient variation across the measured dimensions. Because all items were measured using a seven-point Likert scale, the possible score range was from 1 to 7.

**Table 5:** Descriptive statistics of first-order dimensions.

Construct / Dimension	Mean	SD
Pleasantness	3.273	0.987
Harmony	3.361	0.974
Eventfulness	3.427	0.99
Objective Authenticity	3.693	0.874
Existential Authenticity	3.661	0.992
Cultural Congruence	3.532	0.863
Being Away	3.467	0.913
Fascination	3.474	0.855
Extent	3.388	0.885
Compatibility	3.340	0.820

To better integrate the acoustic measurements with the survey results, site-specific descriptive results are reported in Table 6. For each sampling point, the table presents LAeq, sample size, and mean scores for positive heritage soundscape perception, cultural authenticity, and restorative experience. These results are not intended to replace the individual-level SEM, but they help explain how objective acoustic conditions and perceived soundscape qualities varied across the ten locations.

**Table 6:** Site-specific acoustic and perceptual characteristics across ten sampling points.

Sampling point (category)	Sample Size (n)	LAeq [dB(A)]	Positive SP Mean	CA Mean	RE Mean
Baoguo Temple (Religious)	52	54.2	3.419	3.692	3.397
Fuhu Temple (Mixed)	50	48.5	3.413	3.547	3.427
Qingyin Pavilion (Mixed)	48	61.8	3.491	3.509	3.294
Ecological monkey area (Natural)	49	58.4	3.102	3.490	3.452
Wannian Temple (Religious)	51	52.6	3.397	3.641	3.497
Hongchunping (Mixed)	53	46.7	3.407	3.757	3.413
Jiulinggang trail (Natural)	55	43.5	3.384	3.683	3.415
Xixiangchi (Mixed)	54	49.2	3.358	3.660	3.244
Leidongping to Jieyin Hall trail (Natural)	46	47.8	3.307	3.399	3.271
Golden Summit (Religious)	44	56.3	3.230	3.646	3.357

*Note: Positive SP = positive heritage soundscape perception; CA = cultural authenticity; RE = restorative experience.*

### 4.2 Measurement model

As shown in Table 7, the Cronbach’s *α* values for all first-order constructs ranged from 0.766 to 0.883, while the composite reliability values ranged from 0.784 to 0.884. All values exceed the recommended threshold of 0.70, indicating good internal consistency. The AVE values ranged from 0.509 to 0.694, all above the recommended threshold of 0.50, which supports convergent validity. In addition, the standardized factor loadings of the retained indicators ranged from 0.697 to 0.855.

**Table 7:** Construct reliability and convergent validity.

Second-order Constructs	First-order Dimensions	Items	First-order Loadings	Second-order Loadings	*α*	CR	AVE
**Positive Heritage Soundscape Perception**	Pleasantness	SPP1	0.793	0.746	0.856	0.858	0.666
		SPP2	0.855				
		SPP3	0.799				
	Harmony	SPH1	0.833	0.863	0.872	0.872	0.694
		SPH2	0.827				
		SPH3	0.839				
	Eventfulness	SPE1	0.817	0.795	0.85	0.85	0.653
		SPE2	0.819				
		SPE3	0.789				
**Cultural Authenticity**	Objective Authenticity	CAOA1	0.762	0.736	0.784	0.784	0.547
		CAOA2	0.732				
		CAOA3	0.724				
	Existential Authenticity	CAEA1	0.805	0.791	0.834	0.833	0.626
		CAEA2	0.774				
		CAEA3	0.794				
	Cultural Congruence	CACC1	0.743	0.757	0.792	0.792	0.56
		CACC2	0.764				
		CACC3	0.738				
**Restorative Experience**	Being Away	REBA1	0.773	0.736	0.883	0.884	0.654
		REBA2	0.841				
		REBA3	0.804				
		REBA4	0.815				
	Fascination	REF1	0.745	0.822	0.858	0.859	0.602
		REF2	0.795				
		REF3	0.771				
		REF4	0.793				
	Extent	REE1	0.784	0.787	0.857	0.857	0.6
		REE2	0.778				
		REE3	0.75				
		REE4	0.785				
	Compatibility	REC1	0.768	0.763	0.791	0.794	0.562
		REC2	0.781				
		REC4	0.697				

Although REC4 was slightly below the conventional 0.70 threshold, it was retained because the compatibility construct maintained acceptable reliability and AVE, and the item was theoretically relevant. Item REC3 was removed during the measurement model refinement process due to its low factor loading (0.328). The second-order loadings ranged from 0.736 to 0.863, indicating that the first-order dimensions adequately reflected their corresponding higher-order constructs.

Discriminant validity was then examined using the HTMT. As shown in Table 8, all HTMT point estimates were below the conservative threshold of 0.85, with the highest value being 0.692 between Harmony and Eventfulness. In addition, the 95% bootstrap confidence intervals for all HTMT values did not include 1.00, providing further support for discriminant validity. This additional inference-based assessment strengthens the evidence that the constructs are empirically distinct.

**Table 8:** Discriminant validity (HTMT).

Constructs	1	2	3	4	5	6	7	8	9	10
1. Being away										
2.Compatibility	0.531									
3. Cultural Congruence	0.488	0.426								
4. Eventfulness	0.336	0.33	0.178							
5. Existential Authenticity	0.449	0.425	0.612	0.201						
6. Extent	0.589	0.656	0.393	0.347	0.411					
7. Fascination	0.592	0.631	0.453	0.395	0.465	0.645				
8. Harmony	0.402	0.423	0.283	0.692	0.291	0.423	0.484			
9. Objective Authenticity	0.423	0.393	0.537	0.195	0.585	0.386	0.495	0.34		
10.Pleasantness	0.347	0.381	0.216	0.612	0.229	0.392	0.408	0.623	0.267	

Note: Bootstrap-based 95% confidence intervals were additionally examined for all HTMT values. The upper bounds of all intervals did not include 1.00, supporting discriminant validity.

### 4.3 Structural model assessment and hypothesis testing

Before interpreting the structural paths, the overall fit of the CB-SEM model was assessed. The results indicated a satisfactory fit between the proposed model and the observed data: *χ²* = 543.832, *df* = 514, *χ²/df* = 1.058, *p* = 0.175, RMSEA = 0.011, 90% CI [0.000, 0.019], SRMR = 0.034, GFI = 0.941, AGFI = 0.931, NFI = 0.940, TLI = 0.996, and CFI = 0.996. The non-significant chi-square value, low RMSEA and SRMR values, and high incremental fit indices indicate that the model fit was adequate for further structural path analysis.

After establishing acceptable overall fit, the structural relationships were examined. Positive heritage soundscape perception was significantly and positively associated with cultural authenticity (*β* = 0.405, SE = 0.056, *t* = 7.181, *p* < 0.001), supporting H1. Cultural authenticity was significantly and positively associated with restorative experience (*β* = 0.568, SE = 0.046, t = 12.432, *p* < 0.001), supporting H2. In addition, positive heritage soundscape perception was significantly and positively associated with restorative experience (*β* = 0.396, SE = 0.050, *t* = 7.883, *p* < 0.001), supporting H3.

The squared multiple correlations showed that positive heritage soundscape perception explained 16.4% of the variance in cultural authenticity, while positive heritage soundscape perception and cultural authenticity together explained 66.2% of the variance in restorative experience. Although R² is not the primary fit criterion in covariance-based structural equation modeling, these values still provide useful information about the explanatory power of the proposed model.

**Fig 3:**
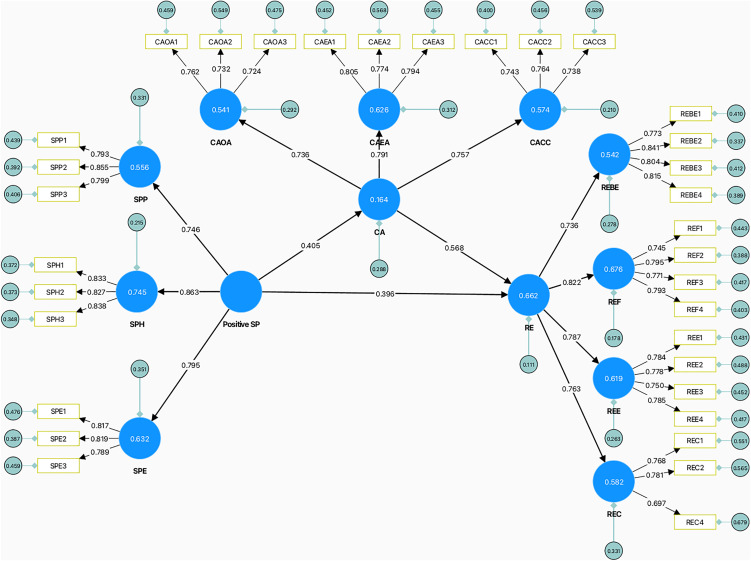
Structural model results. The figure was created by the authors based on the SEM results of the present study and does not include third-party copyrighted content. ***Note:*** *Values on the arrows are standardized path coefficients. Values inside the circles are R² values.*

The indirect association of positive heritage soundscape perception with restorative experience through cultural authenticity was also significant (β = 0.230, SE = 0.042, t = 4.869, p < 0.001, 95% bootstrap confidence interval [0.134, 0.292]). Because both the direct effect and the indirect effect remained significant, cultural authenticity was found to play a partial mediating role in the relationship between positive heritage soundscape perception and restorative experience, thus supporting H4. The standardized structural paths and R² values are presented in Fig 3.

### 4.4 Mediation analysis

The mediating role of cultural authenticity was further examined using bootstrap-based indirect effect estimation. As shown in Table 9, the indirect effect of positive heritage soundscape perception on restorative experience through cultural authenticity was significant (*β* = 0.230, SE = 0.042, *t* = 4.869, *p* < 0.001, 95% CI [0.134, 0.292]). Because the confidence interval did not include zero and the direct effect of positive heritage soundscape perception on restorative experience also remained significant, cultural authenticity was identified as a partial mediator of this relationship.

**Table 9:** Results of hypothesis testing.

Hypothesis	Relationship	t-value	*p*	95% CI	Decision
**H1**	Positive SP → CA	7.181	< 0.001	[0.252, 0.504]	Supported
**H2**	CA → RE	12.432	< 0.001	[0.430, 0.714]	Supported
**H3**	Positive SP → RE	7.883	< 0.001	[0.252, 0.465]	Supported
**H4**	Positive SP → CA → RE (Mediation)	4.869	< 0.001	[0.134, 0.292]	Supported

*Note: Positive SP = positive heritage soundscape perception; CA = cultural authenticity; RE = restorative experience.*

### 4.5 Robustness and subgroup analysis

To evaluate the stability of the proposed structural model across major demographic groups, supplementary subgroup analyses were conducted by gender and age. The sample was divided into male (n = 237) and female (n = 265) groups, as well as younger visitors aged 18–35 (n = 260) and older visitors aged 36 and above (n = 242). Because the subgroup analyses were intended as robustness checks rather than formal multigroup SEM tests, standardized path estimates were compared across subgroups to examine whether the main relationship patterns remained consistent.

As shown in Table 10, the three main structural paths remained positive across all subgroups. For both male and female respondents, and for both younger and older visitors, the associations between positive heritage soundscape perception and cultural authenticity (H1), cultural authenticity and restorative experience (H2), and positive heritage soundscape perception and restorative experience (H3) maintained the same direction. These findings provide supplementary evidence for the stability of the proposed framework. However, they should be interpreted as robustness evidence rather than as a substitute for formal multigroup SEM or multilevel modeling.

**Table 10:** Results of the robustness check across gender and age subgroups.

Hypothesized Structural Path	Male Subgroup (n = 237)	Female Subgroup (n = 265)	Younger Group (n = 260)	Older Group (n = 242)
**H1:** Positive SP → CA	0.307	0.287	0.335	0.272
**H2:** CA → RE	0.454	0.382	0.403	0.442
**H3:** Positive SP → RE	0.353	0.370	0.375	0.337

*Note: Positive SP = positive heritage soundscape perception; CA = cultural authenticity; RE = restorative experience.*

## 5 Discussion

This study examined the relationships among positive heritage soundscape perception, cultural authenticity, and restorative experience at Mount Emei. The results showed that positive heritage soundscape perception was associated with restorative experience both directly and indirectly through cultural authenticity. These findings suggest that heritage soundscapes shape visitor restoration not only through sensory qualities, but also through the cultural meanings attached to sound. In other words, sound in sacred heritage settings should not be understood merely as an environmental stimulus. It also functions as a carrier of spiritual identity, historical continuity, and place-based meaning. This perspective is congruent with current research, which shows that heritage soundscapes can influence authenticity, place-related responses, and cultural meaning [16,17,37], while soundscape quality has also been linked to stress recovery in environmental psychology [45]. Importantly, these findings should not be interpreted as suggesting that soundscape perception alone determines restorative experience. Rather, they explain one sound-related pathway within a wider religious and cultural experience shaped by pilgrimage motives, religious atmosphere, spiritual expectations, cultural familiarity, and broader tourism experiences [31–33].

Positive heritage soundscape perception was significantly and positively associated with cultural authenticity (H1 supported, *β* = 0.405). Visitors who perceived the soundscape as pleasant, harmonious, and meaningful were more likely to regard Mount Emei as culturally genuine. This result fits the logic of heritage soundscapes. In a site such as Mount Emei, sounds such as temple bells, chanting, streams, and wind are more than pleasant stimuli. They help visitors interpret the spiritual and historical identity of the place. When these sounds are experienced as fitting the setting, they strengthen the sense that the environment is authentic and culturally grounded. This finding is also in line with recent studies showing that meaningful soundscapes can strengthen heritage value, identity, and sense of place in historic and sacred environments [16,17,37].

At the same time, positive heritage soundscape perception explained 16.4% of the variance in cultural authenticity. This result suggests that sound alone does not fully explain how visitors judge a heritage environment as culturally genuine. Other factors are also likely to matter. Visitors with prior knowledge of Buddhism may respond differently to temple bells and chanting than those without such a background. Spiritual motivation and personal values may shape authenticity perception independently of what visitors hear. Chen et al. [37] similarly found that cultural memory and personal meaning shape sense of place in heritage areas beyond the acoustic environment. In addition, the diversity of visitors at Mount Emei, including domestic tourists, pilgrims, and recreational hikers, means that cultural authenticity may be understood differently across groups. Future studies should therefore explore whether visitor type, cultural background, or religious identity moderates the relationship between positive heritage soundscape perception and cultural authenticity.

Cultural authenticity showed the strongest direct association with restorative experience (H2 supported, β = 0.568). This association was larger than the direct association between positive heritage soundscape perception and restorative experience (β = 0.396). The difference suggests that restoration in a sacred heritage setting is not explained by acoustic quality alone. Visitors appear to recover more deeply when they feel that the cultural environment is genuine and meaningful. This pattern is consistent with Wang’s [20] concept of existential authenticity and also aligns with restoration theory, which links recovery to immersion, fascination, compatibility, and a sense of being away. In Mount Emei, authenticity seems to deepen visitors’ engagement with the environment and strengthen the restorative experience reflected in these components. This interpretation is also consistent with recent work showing that attention restoration can be shaped by multisensory place experience rather than by sound quality alone [2,40,45].

Positive heritage soundscape perception also showed a significant positive direct association with restorative experience (H3 supported, *β* = 0.396). This direct pathway is consistent with earlier findings in environmental psychology and soundscape research [45]. Natural sounds can reduce stress and support effortless attention [47]. In religious environments, culturally meaningful sounds may also produce calmness and attentional focus. At Mount Emei, these two sound types coexist. This may help explain why positive heritage soundscape perception itself showed a relatively strong direct association with restoration. Research on religious historical buildings, rural soundscapes, and natural soundscapes has similarly suggested that positive acoustic evaluations are associated with stronger restorative or comfort-related responses [9,31,45,47].

The site-level descriptive results suggest that visitors’ responses were partly shaped by site-specific acoustic contexts, with variation in acoustic conditions and perceptual evaluations across the ten sampling points. Although LAeq was not modeled as a structural predictor in the present SEM framework, the measured acoustic levels still provide useful contextual information for interpreting the results. Some sampling points with relatively higher LAeq values were still associated with positive perceptual evaluations, which suggests that visitor responses in sacred heritage environments may depend not only on physical loudness, but also on the perceived meaning, appropriateness, and symbolic value of sound. This does not mean that loudness is irrelevant. Rather, it suggests that in culturally charged settings, the experiential meaning of sound cannot be inferred from decibel level alone. Sounds that are physically prominent may still be positively experienced when they are interpreted as natural, sacred, or place-appropriate.

Cultural authenticity played a partial mediating role in the relationship between positive heritage soundscape perception and restorative experience (H4 supported, indirect effect *β* = 0.230, *t* = 4.869). Because the direct association also remained significant, the mediation is partial rather than full. This pattern reveals that heritage soundscapes are associated with restoration through two distinct processes. One pathway is direct: pleasant and harmonious sounds create immediate feelings of calm that support recovery. The other pathway is indirect: meaningful sounds first deepen the visitor's sense that the site is culturally genuine, and that sense of cultural genuineness then enhances restoration. This dual pathway is consistent with the view that psychological restoration in complex environments depends on intermediate cognitive and evaluative processes, not only immediate sensory comfort [4,7,9]. The finding also supports the broader argument that authenticity functions as a psychological bridge in heritage tourism, connecting environmental experience with deeper personal and emotional outcomes [20,28,29].

The physical and spatial characteristics of the sampling points further help illustrate this dual mechanism. At high-altitude religious sites such as the Golden Summit, the acoustic environment includes strong mountain wind, ritual chanting, and dense visitor activity. In such complex conditions, direct sensory comfort may be reduced by crowd noise and environmental intensity. However, chanting and temple bells may still reinforce the sacred identity of the place and strengthen visitors’ perception of authenticity. This, in turn, may support emotional immersion and restoration. Similarly, in places where stream sounds are physically prominent, restoration may still remain high when those sounds are perceived as natural, fitting, and spiritually integrated into the wider heritage setting. These observations suggest that sacred heritage soundscapes may operate through a compensatory experiential logic: cultural meaning can, in some cases, soften or partially offset the potentially negative experience associated with physically intense acoustic conditions. Although this interpretation should be treated cautiously because LAeq was not modeled directly in the SEM, it offers an important direction for future research on heritage soundscapes.

### 5.1 Theoretical implications

This study extends soundscape research into the context of World Heritage tourism. Much of the current soundscape literature focuses on natural parks, forests, and urban green spaces. Comparatively fewer studies have examined sacred heritage sites where natural and cultural sounds coexist within a single experiential setting. By using Mount Emei as the study context, this research shows that positive heritage soundscape perception in heritage environments cannot be understood only through acoustic comfort or naturalness. Cultural meaning also plays an essential role.

The study also contributes to authenticity research by showing that cultural authenticity can function as a mediator between environmental perception and psychological outcome. Earlier tourism studies often treated authenticity mainly as a predictor of satisfaction, loyalty, or memorable experience. The present study extends that role by demonstrating that authenticity also helps explain why a heritage environment may be experienced as restorative. This finding supports the view that authenticity is not only a heritage evaluation concept, but also a psychological mechanism that shapes deeper visitor experience.

Another contribution lies in the extension of restoration theory. Previous restoration research has mainly emphasized natural features such as water, birdsong, and green scenery. The present findings suggest that in sacred heritage environments, culturally meaningful sounds such as temple bells and chanting can also support restoration when they are perceived as authentic and fitting to the place. This broadens the application of restoration theory from predominantly natural environments to culturally embedded and spiritually meaningful heritage settings.

The findings further point to an important implication for heritage acoustics. In conventional environmental management, restoration is often assumed to increase as physical noise decreases. However, the present study suggests that this logic may be incomplete in sacred heritage settings. A sound environment with relatively high physical intensity may still support restoration if the sounds are interpreted as meaningful, appropriate, and culturally authentic. This implies that the experiential and symbolic content of sound may, in some cases, reshape how visitors respond to physical acoustic conditions. Heritage soundscape research should therefore move beyond a purely decibel-based understanding of acoustic quality and pay greater attention to the semantic and cultural payload of sound.

### 5.2 Practical implications

The findings provide several practical implications for heritage site management. Managers of Mount Emei and similar heritage destinations should treat sound as a core part of heritage experience rather than as a background condition. Natural sounds and Buddhist ritual sounds should be protected because they contribute not only to visitor comfort but also to authenticity and restoration. Efforts to reduce intrusive noise from traffic, loudspeakers, or excessive tourist disturbance may help preserve both acoustic quality and cultural atmosphere.

Heritage soundscape management should also be differentiated by zone. In natural zones, the protection of streams, birdsong, and wind-related acoustic qualities may strengthen direct restorative responses. In religious zones, management should preserve the audibility and symbolic value of temple bells, chanting, and ritual activities. In mixed zones, planners should aim for a balanced acoustic experience so that natural and religious sounds complement rather than mask one another. This zoned strategy is especially relevant for Mount Emei because the site contains low-, mid-, and high-altitude environments with different acoustic characters.

It is also important to consider the design of acoustic-ecological buffer zones between highly active visitor areas and core religious spaces. In entrance areas, transport nodes, or crowded transitional zones, natural masking elements such as flowing water, vegetation belts, and wind-responsive bamboo landscapes may be strengthened to reduce the perceptual intrusion of guide broadcasts, crowd noise, or commercial sounds. Such buffer zones would not simply reduce noise. They could also help visitors slow their psychological rhythm and prepare for entry into more contemplative temple environments.

Heritage soundscape planning should further pay attention to the spatiotemporal synchronization of acoustic cues. This means that managers should not only protect individual sounds, but also consider when and where culturally meaningful sounds are most effective. Along demanding routes such as the ascent toward the Golden Summit, where visitor fatigue may increase and natural background noise may become stronger, clear and meaningful ritual sounds may serve as acoustic anchors that reorient attention and reinforce the sacred atmosphere of the destination. In practice, this may involve protecting the transmission range, timing, and perceptual clarity of bells, chanting, or other traditional sound signals, rather than simply maximizing their loudness.

Interpretation and visitor guidance can also be used to strengthen authenticity perception. Information boards, guided soundwalks, digital interpretation tools, and heritage education materials can help visitors understand why certain sounds matter in the history and spiritual life of the site. If visitors better understand the meaning of the sounds they hear, they may be more likely to perceive them as authentic, appropriate, and restorative. This approach is consistent with recent research suggesting that cultural interpretation can enhance the experiential value of heritage soundscapes.

### 5.3 Limitations and future research

This study is subject to several limitations. The research was carried out at only one World Heritage site. Mount Emei is a sacred Buddhist Mountain characterized by a distinctive mix of natural and religious sounds. For this reason, the findings may not readily apply to other heritage settings, such as urban historic districts, archaeological sites, or secular cultural landscapes. Further research is needed to examine whether the proposed model remains valid across a wider range of heritage contexts.

Although Mount Emei is a religious heritage site, this study did not directly measure pilgrimage motivation, religiosity, spiritual expectation, or prior Buddhist knowledge. These factors may shape how visitors interpret religious sounds and how they evaluate cultural authenticity. Future studies should include these variables to examine whether religious motivation moderates the relationship between positive heritage soundscape perception, cultural authenticity, and restorative experience.

Another limitation concerns the cross-sectional nature of the data. Although this design is appropriate for testing relationships among constructs, it does not allow strong causal conclusions to be drawn. Therefore, the directional paths in the proposed SEM should be interpreted as theory-driven statistical associations rather than causal effects. Future studies are encouraged to adopt longitudinal or experimental research designs to test these relationships and underlying mechanisms more directly.

In addition, this study has a methodological limitation regarding the role of the objective acoustic data. Although the A-weighted equivalent continuous sound pressure level (LAeq) was measured at each sampling point, it was used mainly as contextual environmental information rather than as a modeled variable in the SEM framework. As a result, the present study cannot make direct statistical inferences about how physical sound pressure levels relate to visitors’ subjective soundscape perception and restorative experience. In addition, the questionnaire responses were nested within 10 sampling sites, whereas the analysis was conducted at a single level. Future studies may incorporate objective acoustic indicators more directly into the analytical model and, where possible, apply multilevel modeling to better distinguish environmental-level and individual-level influences.

## Supporting information

S1 Table Bilingual questionnaire items, including the Mandarin and English versions of all measurement items. (DOCX)

S1 Data Anonymized dataset and codebook used to reproduce the descriptive statistics, reliability and validity tests, SEM analysis, and robustness checks. (CSV)
