# Supplementary material for: How soundscape shapes restorative experience in a world heritage site: The mediating role of cultural authenticity at Mount Emei, China
Source: PLoS One. 2026 Aug 7;21(8):e0354957. doi: 10.1371/journal.pone.0354957 (PMC13450790; doi:10.1371/journal.pone.0354957)
Supplement: Supplementary file 1 [file pone.0354957.s001.docx]

**S1 Table.** Bilingual questionnaire items, including the Mandarin and English versions of all measurement items.

| **Construct** | **Dimension** | **Item** | **References** |
| --- | --- | --- | --- |
| **Positive Heritage Soundscape Perception (Positive SP)** | Pleasantness | SPP1: The sounds I hear here are pleasant.  SPP1：我在这里听到的声音令人愉悦。  SPP2: The soundscape at this location makes me feel comfortable.  SPP2：这个地点的声景让我感到舒适。  SPP3: The overall sound environment here is agreeable.  SPP3：这里整体的声音环境是令人满意的。 | [1,2] |
|  | Harmony | SPH1: The different sounds here fit well together.  SPH1：这里不同类型的声音能够很好地融合在一起。  SPH2: The sounds at this location feel balanced and harmonious.  SPH2：这个地点的声音让我感到平衡且和谐。  SPH3: The sounds here do not seem out of place.  SPH3：这里的声音并不显得突兀。 |  |
|  | Eventfulness | SPE1: The soundscape here is varied and interesting.  SPE1：这里的声景丰富多样且有趣。  SPE2: There are many different types of sounds to hear at this location.  SPE2：在这个地点可以听到许多不同类型的声音。  SPE3: The sounds here change and give a sense of activity.  SPE3：这里的声音会发生变化，并带来一种活动感。 |  |
| **Cultural Authenticity (CA)** | Objective Authenticity | CAOA1: The religious sounds at Mount Emei (e.g., temple bells, chanting) feel historically genuine.  CAOA1：峨眉山的宗教声音（如寺庙钟声、诵经声）让我感到具有历史真实性。 | [3,4] |
|  |  | CAOA2: The cultural practices and sounds I experienced at Mount Emei appear to be well preserved.  CAOA2：我在峨眉山体验到的文化活动和声音让我感觉其传统特色得到了较好保留。 |  |
|  |  | CAOA3: The sounds I experienced at Mount Emei reflect the heritage of Buddhist culture.  CAOA3：我在峨眉山体验到的声音反映了佛教文化遗产特色。 |  |
|  | Existential Authenticity | CAEA1: Experiencing the soundscape here helps me feel more connected to my inner self.  CAEA1：体验这里的声景有助于我与内在自我产生更深的连接。 |  |
|  |  | CAEA2: Being in this acoustic environment makes me feel a deeper sense of personal meaning.  CAEA2：置身于这种声音环境中，让我感受到更深层的个人意义。 |  |
|  |  | CAEA3: The sounds here create a sense of stepping into a different and more meaningful world.  CAEA3：这里的声音营造出一种进入不同且更有意义世界的感觉。 |  |
|  | Cultural Congruence | CACC1: The soundscape at this site matches my expectations of what an authentic Buddhist heritage site should sound like.  CACC1：这个地点的声景符合我对真实佛教遗产地声音特征的期待。 |  |
|  |  | CACC2: The acoustic environment here fits the cultural and spiritual character of the site.  CACC2：这里的声音环境与该地点的文化和精神特征相契合。 |  |
|  |  | CACC3: The overall sound atmosphere is consistent with the cultural identity of Mount Emei.  CACC3：整体声音氛围与峨眉山的文化身份一致。 |  |
| **Restorative Experience (RE)** | Being Away | REBA1: The sounds here help me forget about the demands of everyday life.  REBA1：这里的声音帮助我暂时忘记日常生活的压力和要求。 | [5,6] |
|  |  | REBA2: Listening to this soundscape makes me feel mentally distant from my daily routine.  REBA2：聆听这里的声景让我在心理上远离日常生活。 |  |
|  |  | REBA 3: The sonic environment at this location gives me a sense of escape from ordinary pressures.  REBA3：这个地点的声音环境让我产生逃离日常压力的感觉。 |  |
|  |  | REBA 4: When I listen to the sounds here, my mind feels free from the concerns of daily life.  REBA4：当我聆听这里的声音时，我的思绪会从日常生活的烦恼中释放出来。 |  |
|  | Fascination | REF1: I find the sounds at this location naturally interesting and engaging.  REF1：我觉得这个地点的声音自然地令人感兴趣并吸引人。 |  |
|  |  | REF2: The soundscape here captures my attention without any effort on my part.  REF2：这里的声景能够毫不费力地吸引我的注意力。 |  |
|  |  | REF3: I am drawn to listen to the sounds at this site without having to try.  REF3：我会自然而然地被这个地点的声音吸引，而不需要刻意去听。 |  |
|  |  | REF4: The sounds here are fascinating and hold my attention effortlessly.  REF4：这里的声音很有吸引力，并能轻松地保持我的注意力。 |  |
|  | Extent | REE1: The sounds here give me the sense of being part of a larger and richer world.  REE1：这里的声音让我感觉自己是一个更大、更丰富世界的一部分。 |  |
|  |  | REE2: The soundscape at this location feels coherent and complete, like a whole sound world.  REE2：这个地点的声景让我感到连贯且完整，仿佛是一个整体的声音世界。 |  |
|  |  | REE3: The sounds here create the feeling that there is much more to explore and experience.  REE3：这里的声音让我感觉还有更多内容可以探索和体验。 |  |
|  |  | REE4: The sonic environment makes the place feel spacious and deeply immersive.  REE4：这里的声音环境使这个地方显得开阔且具有深度沉浸感。 |  |
|  | Compatibility | REC1: The soundscape here matches what I was hoping to experience at this site.  REC1：这里的声景符合我希望在这个地点获得的体验。 |  |
|  |  | REC2: The sounds at this location are well suited to the kind of experience I was looking for.  REC2：这个地点的声音非常适合我所期待的体验类型。 |  |
|  |  | REC3: I feel that the soundscape here supports the activities and feelings I want to have.  REC3：我觉得这里的声景支持我想要进行的活动和获得的感受。 |  |
|  |  | REC4: The sonic environment fits naturally with my needs and desires at this moment.  REC4：这里的声音环境自然地符合我此刻的需求和愿望。 |  |

***Note:*** *All items were rated on a seven-point Likert scale ranging from 1 = strongly disagree to 7 = strongly agree. Item REC3 was removed during measurement model refinement due to its low factor loading (0.328). The questionnaire was originally administered in Mandarin Chinese, and the English versions are provided for publication and replication purposes.*

**Reference**

1.	Axelsson Ö, Nilsson ME, Berglund B. A principal components model of soundscape perception. J Acoust Soc Am. 2010;128: 2836–2846. doi:10.1121/1.3493436

2.	International Organization for Standardization. ISO/TS 12913-2:2018. In: ISO [Internet]. 2018 [cited 8 Mar 2026]. Available: https://www.iso.org/standard/75267.html

3.	Cohen E. Authenticity and commoditization in tourism. Annals of Tourism Research. 1988;15: 371–386. doi:10.1016/0160-7383(88)90028-X

4.	Wang N. Rethinking Authenticity In Tourism Experience. The Political Nature of Cultural Heritage and Tourism. Routledge; 1999. pp.469-490.

5.	Hartig T, Korpela K, Evans GW, Gärling T. A measure of restorative quality in environments. Scandinavian Housing and Planning Research. 1997;14: 175–194. doi:10.1080/02815739708730435

6.	Qiu M, Jin X, Scott N. Sensescapes and attention restoration in nature-based tourism: Evidence from China and Australia. Tourism Management Perspectives. 2021;39: 100855. doi:10.1016/j.tmp.2021.100855
