# Supplementary material for: How soundscape shapes restorative experience in a world heritage site: The mediating role of cultural authenticity at Mount Emei, China
Source: PLoS One. 2026 Aug 7;21(8):e0354957. doi: 10.1371/journal.pone.0354957 (PMC13450790; doi:10.1371/journal.pone.0354957)
Supplement: Supplementary file 2 [file pone.0354957.s004.docx]

Dear Academic Editor and Reviewers,

We sincerely thank the Academic Editor and both reviewers for their valuable comments and constructive suggestions. We have carefully revised the manuscript entitled “How Soundscape Shapes Restorative Experience in a World Heritage Site: The Mediating Role of Cultural Authenticity at Mount Emei, China” and addressed all comments point by point. We believe these revisions have improved the clarity, methodological transparency, and overall quality of the manuscript.

Below, we provide a detailed response to each comment.

**Response to the Academic Editor**

**Comment 1:**
Please ensure that the manuscript follows PLOS ONE formatting requirements.

**Response:**
We checked the manuscript structure and prepared the required revision package. The revised submission will include a separate Response to Reviewers file, a marked-up manuscript with changes highlighted, and an unmarked manuscript file using the journal's requested file labels.

**Comment 2:**
Please provide additional details regarding participant consent and ethics.

**Response:**
We expanded the ethics statement in Section 3.2. The revised manuscript now explicitly states that informed written/electronic consent was obtained through a consent checkbox before participation. We also clarified that all participants were adults aged 18 years or above and that no personally identifiable information was collected. In addition, we explained the legal basis for waiving formal ethical approval according to Article 32 of the Ethical Review Measures for Life Sciences and Medical Research Involving Humans in China.

**Comment 3 and 4:**
Please revise the Data Availability Statement and provide access to the anonymized dataset.

Response:
We revised the Data Availability Statement and uploaded the anonymized dataset, codebook, and full questionnaire as Supporting Information files. The revised manuscript now clearly states that the dataset underlying the findings is publicly available without restriction.

**Comment 5 and 6:**
Please revise the authorship information.

**Response:**
The authorship information in both the manuscript file and submission system has been revised accordingly.

**Comment 7:**
Figure 2 may contain copyrighted map or satellite imagery.

**Response:**
We revised the Figure 2 caption to clarify that the figure was created by the authors using the authors' sampling-point coordinates and public-domain Natural Earth boundary data. The caption now states that no proprietary map, satellite, Google Maps, Google Earth, or Street View imagery was used.

**Comment 8:**
Figure 3 may contain copyrighted content.

**Response:**
Figure 3 was regenerated by the authors using original SEM results from the present study. The revised caption explicitly states that the figure does not contain third-party copyrighted material.

**Comment 9:**
Please ensure that you refer to Figure 3 in your text as, if accepted, production will need this reference to link the reader to the figure.

**Response:**
A direct reference to Figure 3 has been added in Section 4.3.

**Response to Reviewer #1**

**Comment 1:**
Theoretical framework: the relationship between natural and religious sounds and restorative experience is well known and well documented in the literature. Why do the authors want to investigate the relationships? Of course, there is a positive relationship. However, the soundscape experience is not the only factor in the restorative experience at this cultural heritage site. It is a religious site. The main motivation for visiting this area is pilgrimage and religious motives, not just religious sounds. Also, the restorative experience or tourism depends on many factors, not just sound. Please look into the tourism literature or religious tourism literature again.

**Response:**
We agree that restorative experience in a religious World Heritage context is influenced by multiple factors beyond sound alone, including pilgrimage motives, spiritual expectations, cultural familiarity, and broader tourism experiences. To address this concern, we revised the Introduction, Literature Review, Discussion, and Limitations sections. In the Introduction, we clarified that the present study does not attempt to explain restorative experience exclusively through soundscape perception. We added that restorative experience in sacred heritage settings is shaped by multiple psychological, cultural, and tourism-related factors, while this study specifically focuses on soundscape perception as one important experiential component within this broader context.

We also expanded the literature review to discuss the role of pilgrimage motives, spiritual expectations, prior religious knowledge, and cultural familiarity in shaping visitors’ interpretation of religious sounds. In the Discussion, we further emphasized that sound in sacred heritage settings should not be understood merely as an acoustic stimulus, but also as a carrier of spiritual identity, historical continuity, and place-based meaning. In the Limitations section, we acknowledged that pilgrimage motivation, religiosity, spiritual expectation, and prior Buddhist knowledge were not directly measured and should be examined in future studies.

Therefore, the revised manuscript positions soundscape perception as one meaningful pathway within a broader religious and cultural tourism experience, rather than as the sole determinant of restorative experience.

**Comment 2:**
There is a sufficient information about the data collection. I think it would be good to document the university's ethical approval of the research design. Also, document the questionnaire in Mandarin and English in the appendix (or online appendix). Can you explain why you have only asked domestic visitors or those who understand Mandarin? Can you add a sentence explaining why international visitors are not included in this survey? I also suggest making the data available to allow replication (on an online archive).

**Response:**
We expanded the Methods section to include an Ethics statement specifying informed written/electronic consent and the ethics-waiver rationale. We added the full questionnaire in bilingual form as Appendix A and Supporting Information. We also added a paragraph explaining that the survey was administered in Mandarin because the study focused on Chinese visitors, who constitute the main visitor group, and because including international visitors would introduce additional heterogeneity in language, cultural familiarity, and travel motivations. We revised the Data Availability Statement and will provide the anonymized dataset and codebook as Supporting Information.

**Comment 3:**
“ Likert scale: 1 = strongly disagree, 7 = strongly agree. “

I appreciate that the authors elaborate on common-method bias and report the discriminant validity measures and all other validity measures. However, the authors must also present descriptive statistics for the Likert-scale variables. I do not find these statistics. It is important to show the means and the standard deviation to see that there is enough variation in the data

**Response:**

We added a new descriptive statistics table (Table 5) reporting the mean and standard deviation for all first-order dimensions. We also added a descriptive interpretation in Section 4.1 to demonstrate that the Likert-scale responses showed sufficient variation across the measured constructs.

**Comment 4:**
Table 6. Results of hypothesis testing

Do not show both the standard errors and the t-stat. One is redundant.

**Response:**

We revised the hypothesis testing table to reduce redundant statistical reporting. Specifically, the original Table 6 has been renumbered as Table 9 in the revised manuscript. Following the reviewer’s recommendation, the standard error column was removed, and the revised table now reports only the key hypothesis-testing information, including the t-value, p-value, 95% confidence interval, and decision. The standardized path coefficients are presented in the text and Figure 3 to avoid unnecessary repetition.

**Comment 5:**
“After establishing acceptable overall fit, the structural relationships were examined. SP had a significant positive effect on CA (β = 0.405, SE = 0.056, t = 7.181, p < 0.001), supporting H1. CA also had a significant positive effect on RE (β = 0.567, SE = 0.046, t = 12.432, p < 0.001), supporting H2. In addition, SP had a significant positive direct effect on RE (β = 0.396, SE = 0.050, t = 7.883, p < 0.001), supporting H3.”

I suggest that the authors should be more careful in interpreting the estimated coefficients. Using CB-SEM and cross-sectional data, you will not obtain effects or impacts. You should interpret the estimated coefficient as a relationship or association.

**Response:**

We appreciate this important methodological clarification. Following the reviewer’s suggestion, we carefully revised the manuscript to avoid causal wording that could overstate the interpretation of cross-sectional CB-SEM results. Terms such as “effect,” “impact,” “influence,” and “determine” were revised to more appropriate relational expressions such as “association,” “relationship,” and “positively associated with.” These revisions were made throughout the manuscript, particularly in the Introduction, Literature Review, Results, and Discussion sections.

**Comment 6:**
Another point concerns the normality assumption in CB-SEM. I do not find the test. I also suggest that authors justify their choice between CB-SEM and PLS-SEM.

**Response:**

We appreciate this important suggestion. To address the normality assumption, a new normality assessment was added in Section 3.5 and Table 4. The revised manuscript now reports univariate skewness, univariate kurtosis, and Mardia’s multivariate kurtosis results. The univariate skewness values ranged from -0.082 to 0.213, and the univariate kurtosis values ranged from -0.586 to 0.203, both within commonly accepted thresholds. Mardia’s multivariate kurtosis also did not indicate severe multivariate non-normality (z = -1.480, p = 0.139). These results suggest that the data were acceptable for CB-SEM estimation.

We also clarified the rationale for using CB-SEM rather than PLS-SEM. CB-SEM was selected because the present study aimed to test a theory-driven reflective measurement model and evaluate overall model fit, rather than primarily maximize prediction. In addition, the sample size and distributional characteristics were considered suitable for CB-SEM estimation.

**Comment 7:**
I know that most scholars never conduct robustness checks when using structural equation modelling. I think given that you have a relatively large sample size you can think of some robustness checks. Maybe you can provide separate estimation results for women or for men. Alternatively, provide results for younger people versus older people.

**Response:**

Following the reviewer’s suggestion, additional robustness analyses were conducted across gender and age subgroups. The results are presented in Section 4.5 and Table 10. The subgroup analyses showed that the main structural path directions remained positive and generally stable across male and female respondents as well as younger and older visitor groups. These analyses were conducted as supplementary robustness checks rather than formal multigroup SEM tests.

**Comment 8:**

Please reduce the number of abbreviations in the text.

**Response:**

We carefully reduced unnecessary abbreviations throughout the manuscript. In particular, the full terms “soundscape perception,” “cultural authenticity,” and “restorative experience” are now provided at the first mention in each major section or subsection, followed by the abbreviations SP, CA, and RE where appropriate. We also avoided using excessive first-order construct abbreviations in the main text and retained such abbreviations mainly in tables and figures for clarity.

**Response to Reviewer #2**

**Comment 1:**
First, I think the contribution of acoustic data is not clear. I appreciate table 1, but acoustic data are not integrated in the model and do not concur to answer research questions. I think you could think about how to better integrate acoustic data in the results.

**Response:**

To better integrate the acoustic data into the study, we revised the Results and Discussion sections. Specifically, a new site-level descriptive comparison table (Table 6) was added in Section 4.1, presenting LAeq values together with sample size and the mean scores of soundscape perception, cultural authenticity, and restorative experience across the ten sampling points.

We also clarified in Section 3.3 that the acoustic measurements were used as contextual environmental information rather than latent SEM variables, because LAeq was measured at the site level whereas the SEM focused on individual-level perceptions. In the Discussion section, we further explained how the site-level acoustic results help contextualize the perceptual findings. In particular, we noted that some locations with relatively higher LAeq values were still associated with positive perceptual evaluations, suggesting that in sacred heritage settings, visitors’ responses depend not only on physical loudness but also on the perceived meaning, appropriateness, and symbolic value of sound.

**Comment 2:**
Second, the paper lacks a descriptive analysis of the survey results, and especially it does not say anything about the perceived differences between the sites, which, in my opinion are quite interesting considering your final discussion. Site-specific responds could integrate table 1. Furthermore, it would be interesting to know more about respondents: are they religious? Did you find any gender or age-related differences in the answers? I would recommend to add all the questions of the questionnaire in Annex 1, not only the constructs.

**Response:**

Thank you for this valuable suggestion. First, descriptive statistics were added in Section 4.1 and Table 5. Second, to address the reviewer’s recommendation regarding site-specific responses, a new site-level comparison table (Table 6) was introduced. This table integrates the acoustic measurements previously summarized in Table 1 with the corresponding mean scores of soundscape perception (SP), cultural authenticity (CA), and restorative experience (RE) across the ten sampling points, thereby allowing clearer comparison of perceptual differences between sites.

Third, additional subgroup robustness analyses based on gender and age were added in Section 4.5 and Table 10. Finally, the full questionnaire, including demographic questions, screening items, and all measurement items, has been provided in Appendix A in both English and Mandarin Chinese.

**Comment 3:**
Third, the paper is too focused on the SEM, which present some methodological issues. Although it is well structured and clearly conceptualized the SEM, I would like to point out some issues. The reported fit indices (RMSEA = 0.000; CFI = 1.000; TLI = 1.000) are unusually perfect for a model of this complexity and raise concerns about potential overfitting or estimation issues. Further clarification of the estimation procedure and model specification is required. The hierarchical structure of the data (participants nested within locations) is not accounted for, which may bias inference.

**Response:**

Thank you for this important observation. We agree that the previously reported fit indices were unusually high for a model of this complexity. Following the reviewer’s suggestion, we re-examined the measurement model specification and estimation procedure.

During the measurement model refinement process, item REC3 was removed because of its low standardized factor loading (0.328). Item REC4 was retained because, although its loading was slightly below the conventional 0.70 threshold, the compatibility construct maintained acceptable reliability and AVE, and the item was theoretically relevant. The SEM was then re-estimated using the refined measurement model.

As a result, the updated fit indices were less extreme and more consistent with the complexity of the model: χ² = 543.832, df = 514, χ²/df = 1.058, p = 0.175, RMSEA = 0.011, SRMR = 0.034, TLI = 0.996, and CFI = 0.996. These revised results have been updated in Sections 4.2 and 4.3, Table 7, and Figure 3. The main structural relationships remained significant and consistent with the proposed hypotheses.

Regarding the hierarchical structure of the data, we acknowledge that respondents were recruited across ten sampling locations. However, because the study included only ten sampling points, full multilevel SEM was not considered sufficiently stable for the present analysis. We therefore treated LAeq as site-level contextual information and conducted the SEM at the individual perception level. To improve transparency, we clarified this analytical decision in Section 3.3 and added site-level descriptive comparisons in Section 4.1 and Table 6. We also acknowledged this limitation in the Limitations section and recommended future studies to apply multilevel modeling when a larger number of sampling sites is available.

**Comment 4:**
Four, the mediation analysis is statistically appropriate; however, the results are interpreted in causal terms despite the cross-sectional design. Without temporal or experimental evidence, the proposed directional relationships (SP → CA → RE) should be interpreted as associations rather than causal effects.

**Response:**

We appreciate this important methodological clarification. We agree that the cross-sectional SEM design does not allow strong causal conclusions. Therefore, we carefully revised the manuscript to avoid causal wording that could overstate the interpretation of the findings.

Terms such as “effect,” “impact,” “influence,” and “determine” were replaced, where appropriate, with relational expressions such as “association,” “relationship,” “linked with,” and “positively associated with.” These revisions were made throughout the manuscript, particularly in the Introduction, Literature Review, Hypothesis Development, Results, Discussion, and Limitations sections.

We also added a clear statement in the Limitations section that the directional paths in the SEM should be interpreted as theory-driven statistical associations rather than causal effects.

**Comment 5:**
Finally, some minor observations are about the survey methodology. How did you distribute the questionnaire? Were they paper questionnaries? How many researchers were involved in the distribution? Did you distribute the questionnaire at the beginning of the path or in each sampling point? In which period of the day? How long is the route to from sample point 1 to the last sample point?

**Response:**

Additional methodological details were added to Section 3.2. The revised manuscript now clarifies that both paper-based and QR-code-based questionnaires were distributed by four trained research assistants at individual sampling points rather than only at the entrance of the route. The survey was conducted during regular daytime visiting hours on both weekdays and weekends.

Participants were invited to complete the questionnaire after spending at least five minutes within the local sound environment so that their responses could be linked to the corresponding acoustic context. Because respondents were recruited at individual sampling points rather than after completing the entire tourist route, the analysis focused on site-specific acoustic exposure rather than full-route walking experience.

We sincerely thank the Academic Editor and Reviewers again for their constructive feedback. We believe that the revised manuscript has been substantially strengthened and hope that the revised manuscript is now suitable for further consideration by PLOS ONE.

Sincerely,

Haoran Wang, on behalf of all authors
