# Supplementary material for: How soundscape shapes restorative experience in a world heritage site: The mediating role of cultural authenticity at Mount Emei, China
Source: PLoS One. 2026 Aug 7;21(8):e0354957. doi: 10.1371/journal.pone.0354957 (PMC13450790; doi:10.1371/journal.pone.0354957)
Supplement: Supplementary file 3 [file pone.0354957.s005.docx]

**Response to Reviewers**

We sincerely thank the Editor and both Reviewers for their careful reading of our manuscript and their constructive comments. We have addressed each point raised and believe the revised manuscript is substantially improved. All changes are described in detail below.

**Response to Reviewer #1**

**Comment 1:**

The hypotheses are generic

H1: Soundscape perception is significantly and positively associated with cultural authenticity

To be read as

H1: The perception of a pleasant soundscape is significantly and positively correlated with cultural authenticity

H3: Soundscape perception is significantly and positively associated with restorative experience.

Of course, a pleasant soundscape is closely linked to a relaxing experience.

Why do you want to investigate this?

The theoretical model is not convincing.

All three dimensions are outcome variables: scoundscape experience, cultural authenticity and restorative experience

**Response:**

Thank you for this important comment. We agree that the previous wording of “soundscape perception” was too broad and could imply a general or neutral perception of sound. We have therefore revised the focal construct of the present study as “positive heritage soundscape perception” or “visitors’ positive perception of the heritage soundscape.” We now explicitly define this construct as visitors’ positive evaluation of the heritage soundscape in context, reflected by perceived pleasantness, harmony, and eventfulness. The hypotheses have also been rewritten so that H1 and H3 no longer refer to generic soundscape perception. In addition, we revised the theoretical model explanation to clarify that the three constructs are not treated as equivalent outcomes. Positive heritage soundscape perception is now positioned as visitors’ appraisal of acoustic environmental cues, cultural authenticity as a meaning-based interpretive mechanism, and restorative experience as the psychological outcome. We also clarified why the direct association between positive soundscape evaluation and restoration is tested: it provides a comparison with the indirect pathway through cultural authenticity and allows us to examine whether cultural meaning explains restoration beyond immediate sensory comfort. These revisions appear in the Abstract, Introduction, Sections 2.4, 2.5.1, 2.5.3, 2.5.5, the hypotheses, Figure 1, the Results, and the Discussion.

Specifically, H1 was revised as: “Visitors’ positive perception of the heritage soundscape, reflected by pleasantness, harmony, and eventfulness, is positively associated with perceived cultural authenticity.” H3 was revised as: “Visitors’ positive perception of the heritage soundscape is positively associated with restorative experience.” We also added a new explanation in Section 2.5.5 to clarify that positive heritage soundscape perception is treated as an environmental appraisal, cultural authenticity as a meaning-based interpretive mechanism, and restorative experience as the psychological outcome.

**Comment 2:**

Figure 1. Proposed research model

What is the source? Line 82

**Response:**

Thank you for pointing this out. We have revised Figure 1 and its caption. The figure now identifies the focal construct as “Positive Heritage Soundscape Perception” rather than the more generic “Soundscape Perception.” We also added the source statement to the figure caption, explaining that the conceptual model was developed by the authors based on soundscape perception theory, authenticity theory, and restoration theory. The relevant supporting references are now included in the figure caption.

**Comment 3:**

There are too many abbreviations

example

ART proposes that restorative environments help restore attentional resources [4], while SRT emphasizes rapid emotional and physiological recovery from stress [5,19].

**Response:**

We agree and have reduced the use of abbreviations throughout the manuscript. In the theoretical sections and discussion, we now use the full terms “Attention Restoration Theory,” “Stress Reduction Theory,” “positive heritage soundscape perception,” “cultural authenticity,” and “restorative experience” wherever possible. We have removed ART and SRT throughout the manuscript, replacing them with their full terms “Attention Restoration Theory” and “Stress Reduction Theory” at every occurrence.

**Comment 4:**

In my opinion, the Vancouver citation style has not been applied correctly. When the author’s name appears in the text, the reference number should follow immediately after the author’s name.

Similarly, Hong and Jeon showed that contextual and acoustic factors jointly shape soundscape evaluation [9].

To be read as

Similarly, Hong and Jeon [9] showed that contextual and acoustic factors jointly shape soundscape evaluation

**Response:**

Thank you for this correction. We have revised the in-text citations according to the Vancouver style requirement. When authors are named in the sentence, the reference number now follows the author name directly, for example “Hong and Jeon [9] showed…” rather than placing the citation at the end of the sentence. We checked the full manuscript for similar cases and corrected the relevant citations, including references to Axelsson et al., Aletta et al., Hong and Jeon, Jin et al., Hu et al., Mao et al., Wang, Ratcliffe, Bai et al., Yang and Zhang, and others.

**Comment 5:**

Because all latent variables were measured through self-reported questionnaire responses collected in the same survey, common method bias was examined before model evaluation. Harman’s ingle factor test was applied by loading all measurement items into an unrotated exploratory factor analysis. The first unrotated factor accounted for 31.708% of the total variance, which was lower than the commonly used threshold of 50%. On this basis, common method bias was not considered to pose a serious problem in the present study.

The Harman article should be cited. The test is outdated.

**Response:**

Thank you for this helpful comment. We have added the original Harman reference to the reference list and cited it in the common method bias section. We also agree that Harman’s single-factor test is limited and should not be treated as a definitive diagnostic for common method variance. Therefore, we revised the manuscript to clarify that Harman’s test was used only as a preliminary diagnostic, and the result was interpreted cautiously.

To strengthen the assessment, we further added an unmeasured latent method construct (ULMC) approach. In this analysis, all measurement items were allowed to load on both their substantive factors and a common method factor. The results showed that the changes in standardized factor loadings after including the method factor were all below the empirical threshold of 0.20, indicating that common method bias was unlikely to substantially distort the structural estimates. We also cited Podsakoff et al. and Liang et al. to support the limitation of Harman’s test and the use of the ULMC approach. These revisions appear in Section 3.4, Common Method Bias Assessment.

**Response to Reviewer #2**

**Comment:**

I think you could improve the section on cultural authenticity by delving into the academic debate on religious heritage tourism experiences. For instance, the paragraph from line 173 to 179 does not refer to any scientific publication. Another example can be found in the discussion section from line 615 to 617 when you refer to research in rural soundscapes and temple soundscape without citing any scientific reference.

**Response:**

Thank you for these specific observations. We have addressed both instances as follows.

For the paragraph on religious heritage tourism experiences (previously lines 173–179), we have expanded the discussion and added three scientific references. The revised paragraph now discusses how pilgrimage motives, spiritual expectations, prior religious knowledge, and cultural familiarity may shape visitors' interpretation of heritage sounds, and draws on studies of religious historical buildings [27], historically significant places [28], and heritage authenticity [29] to show that sacred environments are experienced through intertwined sensory, cultural, and psychological meanings.

For the discussion passage on rural soundscapes and temple soundscapes (previously lines 615–617), we have added four supporting references. The revised sentence now reads: "Research on religious historical buildings, rural soundscapes, and natural soundscapes has similarly suggested that positive acoustic evaluations are associated with stronger restorative or comfort-related responses [27,33,45,48]." These revisions appear in Section 2.2 and the Discussion, respectively.

We further revised Section 2.2 to clarify that religious heritage tourism experience is not a direct response to environmental stimuli alone, but is constructed through the interaction of sensory cues, cultural knowledge, religious atmosphere, personal motivation, and visitors’ familiarity with the heritage context.

**Comment:**

A more thorough revision of the scientific literature on these themes would enhance the manuscript.

**Response:**

We agree and have conducted a broader revision of the literature across both the theoretical framework and the Discussion. The revised manuscript incorporates additional literature on religious historical buildings, historically significant places, heritage authenticity and well-being, multisensory place experience, rural soundscape restoration, and soundscape-related stress recovery. We believe these additions better situate the study within ongoing academic debates in religious heritage tourism and heritage soundscape research, and strengthen the theoretical basis for the proposed model.
