# Supplementary material for: How soundscape shapes restorative experience in a world heritage site: The mediating role of cultural authenticity at Mount Emei, China
Source: PLoS One. 2026 Aug 7;21(8):e0354957. doi: 10.1371/journal.pone.0354957 (PMC13450790; doi:10.1371/journal.pone.0354957)
Supplement: Supplementary file 4 [file pone.0354957.s006.docx]

**Response to Reviewers**

Dear Academic Editor and Reviewers,

Thank you very much for your careful evaluation of our revised manuscript and for the constructive final comments. We greatly appreciate the positive assessments provided by both reviewers. We have addressed all remaining points raised by Reviewer #1, revised the manuscript accordingly, and ensured that the corresponding changes are highlighted in the revised manuscript. We have also prepared the Supporting Information files requested for replication.

**Response to Reviewer #1**

**Comment 1:**

Which software package is used to estimate CB-SEM?

**Response:**

We have now explicitly specified the software package used for the CB-SEM analysis in Section 3.5, Data Analysis. The revised manuscript now states: “The CB-SEM model was estimated using SmartPLS 4.0.” We also clarified that the bootstrap-based confidence intervals for the mediation analysis were calculated in SmartPLS.

**Comment 2:**

S1 Table. Bilingual questionnaire items

S1 Data. Data

I cannot find the link to the supplementary information. Upload the data to allow replication of the analysis.

**Response:**

We have prepared the Supporting Information files as separate files in the revised submission so that the analysis can be replicated. Specifically, S1 Table contains the bilingual measurement items in both Mandarin Chinese and English, and S1 Data contains the anonymized dataset and accompanying codebook used to reproduce the descriptive statistics, reliability and validity tests, SEM analysis, and robustness checks reported in the manuscript.

We have also clarified the availability and contents of these files in the manuscript. The revised manuscript now states: “The anonymized dataset and accompanying codebook are provided in S1 Data, while the bilingual measurement items are provided in S1 Table.” We also revised the Supporting Information captions at the end of the manuscript to specify the contents of S1 Table and S1 Data.

**Comment 3:**

Introduction

Line 37

Heritage tourism has also grown rapidly in recent years.

An in-text citation is needed here.

Is it really growing?

**Response:**

We agree that the previous statement that heritage tourism “has grown rapidly” was too broad and was not sufficiently supported by the cited evidence. Rather than retaining an overgeneralized growth claim, we removed this wording and replaced it with a more precise, evidence-based statement in the Introduction.

The revised sentence now reads: “Recent research has examined how multisensory experiences in historical and cultural settings can contribute to tourists’ mental restoration [2].”

**Comment 4:**

Line 45

Although these perspectives have been widely applied in forests, parks, and coastal settings, they have been used less often in heritage tourism environments.

Can you add some citations here?

**Response:**

We revised the sentence to describe the relevant previous research settings more precisely and added supporting citations. The revised sentence now reads: “Although these perspectives have been widely applied in natural and nature-based recreational settings, including forests and rural landscapes [1,6–9], they have been used less often in heritage tourism environments.”

**Comment 5:**

Line 167

Although authenticity has been widely studied in relation to visual, architectural, and symbolic features of heritage sites, the role of sound in shaping authenticity perception remains relatively underexplored.

Can you add some citations here:

Widely studied? Which studies

**Response:**

Thank you for this important comment. We agree that the previous wording was too general. We revised the sentence to specify the areas in which authenticity has been examined and added relevant supporting citations. The revised sentence now reads: “Although authenticity has been widely studied in relation to heritage objects, cultural identity, visitor engagement, and heritage consumption [19,20,29,30], the role of sound in shaping authenticity perception remains relatively underexplored.”

In the same paragraph, we retained and clarified the discussion of soundscape-based authenticity by referring to Hu et al. [17], which directly addresses the role of soundscape design in strengthening authenticity perception in historic districts.

**Comment 6:**

Line 812

Although LAeq was measured at each sampling point, it was used mainly as contextual environmental information rather than as a modeled variable in the SEM framework.

Please write out. LAeq: weighted average sound level.

**Response:**

We have now written out the abbreviation using the standard technical term at the relevant occurrence in the Limitations and Future Research section. The revised sentence now reads: “Although the A-weighted equivalent continuous sound pressure level (LAeq) was measured at each sampling point, it was used mainly as contextual environmental information rather than as a modeled variable in the SEM framework.”

Sincerely,

The authors
